# Brassinosteroid Signaling, Crosstalk and, Physiological Functions in Plants Under Heavy Metal Stress

**DOI:** 10.3389/fpls.2021.608061

**Published:** 2021-03-24

**Authors:** Jaspreet Kour, Sukhmeen Kaur Kohli, Kanika Khanna, Palak Bakshi, Pooja Sharma, Arun Dev Singh, Mohd Ibrahim, Kamini Devi, Neerja Sharma, Puja Ohri, Milan Skalicky, Marian Brestic, Renu Bhardwaj, Marco Landi, Anket Sharma

**Affiliations:** ^1^Department of Botanical and Environmental Sciences, Guru Nanak Dev University, Amritsar, India; ^2^Department of Zoology, Guru Nanak Dev University, Amritsar, India; ^3^Department of Botany and Plant Physiology, Czech University of Life Sciences Prague, Prague, Czechia; ^4^Department of Plant Physiology, Slovak University of Agriculture, Nitra, Slovakia; ^5^Department of Agriculture, Food and Environment, University of Pisa, Pisa, Italy; ^6^State Key Laboratory of Subtropical Silviculture, Zhejiang A&F University, Hangzhou, China

**Keywords:** BR biosynthetic pathway, BR signaling, transcription, heavy metal, stress, hormone crosstalk

## Abstract

Brassinosteroids (BRs) are group of plant steroidal hormones that modulate developmental processes and also have pivotal role in stress management. Biosynthesis of BRs takes place through established early C-6 and late C-6 oxidation pathways and the C-22 hydroxylation pathway triggered by activation of the DWF4 gene that acts on multiple intermediates. BRs are recognized at the cell surface by the receptor kinases, BRI1 and BAK1, which relay signals to the nucleus through a phosphorylation cascade involving phosphorylation of BSU1 protein and proteasomal degradation of BIN2 proteins. Inactivation of BIN2 allows BES1/BZR1 to enter the nucleus and regulate the expression of target genes. In the whole cascade of signal recognition, transduction and regulation of target genes, BRs crosstalk with other phytohormones that play significant roles. In the current era, plants are continuously exposed to abiotic stresses and heavy metal stress is one of the major stresses. The present study reveals the mechanism of these events from biosynthesis, transport and crosstalk through receptor kinases and transcriptional networks under heavy metal stress.

## Introduction

Brassinosteroids (BRs) were discovered 40 years ago, and since then an enormous amount of work has been done to illuminate their role in plant physiology (Peres et al., 2019). BRs are poly-hydroxylated steroids that regulate developmental processes such as cell division, cell cycle, elongation, morphogenesis, reproduction, senescence and stress-protective responses (Clouse and Sasse, 1998). They function especially as master switches in triggering the metabolic response to noxious environmental conditions (Bajguz, 2010). BRs are considered as derivatives of 5α-cholesterol, but they vary in structure due to the carbon side-chains. They are present in all parts of plants but are mostly found in seeds and pollen (Bartwal and Arora, 2020). Caño-Delgado et al. (2004) suggested that as animal steroid hormones bind to nuclear receptors that modulate gene expression, BRs also bind to receptors on the cell surface initiating a signal cascade leading to alterations in gene expression. Recent progress in understanding the BR signaling pathways supports the idea that they do not follow a linear path but rather undergo crosstalk with other hormones to combat stress conditions (Nolan et al., 2017). BRs also provide tolerance to plants against abiotic stressors by modulating the activity of enzymatic and non-enzymatic antioxidants (Sharma A. et al., 2016; Sharma et al., 2017; Bartwal and Arora, 2020; Chi et al., 2020; Rattan et al., 2020; Chen et al., 2021). BRs are also reported to stimulate the formulation of phytochelatins as reported by Bajguz, 2002. Reports suggested that brassinolides along with the lead are responsible for the increase in the synthesis of phytochelatins in *Chlorella vulgaris.* To overcome the harmful effects of heavy metals, BRs interact with other hormones like auxin, cytokinin, abscisic acid (ABA), ethylene, and jasmonic acid (JA). In this review we have discussed in detail the molecular mechanism of BR biosynthesis, signaling, and the role of transcriptional networks in the response to heavy metal stress in plants, and the ways that BRs crosstalk with other phytohormones to prevent heavy metal damage.

## Structure and Distribution of BRs

Brassinosteroids share structural resemblance with animal steroids like cholestane, ergo- and stigmastane. The majority of BRs are 5α-cholestane derivatives and structural variation among them emerges because of C-substitutions within the side chains (Ali, 2019). They can be found in all plant parts, yet most of the synthesis occurs in the meristematic zones of seeds and pollen grains. These BR categories represent more than eighty plant species including angiosperms, pteridophytes, algae, and bryophytes (Yokota, 1999). Based on the number of carbon atoms and alkyl groups within side chains, BRs are divided into C-27, -28, and -29 steroids present freely or in conjugation with fatty acids and sugars (Fujioka and Yokota, 2003). Forty to fifty percent of BRs are C-28. The great diversity in the patterns of cyclic groups as well as side chains is mainly responsible for the conversion of BRs into active analogs such as 24-epibrassinolides and 28-homobrassinolides (Kang and Guo, 2011). BRs along with analogs like typhasterol, brassinolide, and castasterone are widely distributed within specialized plant structures like pollens, seeds, flowers, roots, shoots, leaves and stems. The greatest concentration of BRs (100 ng per g fresh weight) have been recorded within pollen grains and seeds compared to 0.01 and 0.1 ng per g fresh weight within shoots and leaves, respectively.

More than 69 different BRs and derivatives have been identified to date from various plants (Bajguz and Tretyn, 2003) including *Castanea crenata* and *Catharanthus roseus*. Along with various BRs there are five conjugates of BRs which are found together with 8 metabolites and 137 analogs of BRs (Liu et al., 2017). The distribution of BRs in various plant families from maximum to minimum runs from angiosperms to gymnosperms, pteridophytes, bryophytes, and algae (Bajguz and Tretyn, 2003). It is well documented that sterols within plants are transformed into brassinolide through teasterone, typhasterol as well as castasterone via the isoprenoid pathway in association with acetyl coA, isopentenyl pyrophosphate (IPP), geranyl pyrophosphate (GPP), mevalonate and farnesyl pyrophosphate (FPP) (Symons et al., 2008). Brassinolides are highly effective forms of BRs that are produced as the end product of BRs synthesis (Zhao and Li, 2012). According to a more limited description, among the metabolites produced during the biosynthesis of BR-lactones, only those that have been formed from 22α and 23α-dihydroxylation would be considered as true BRs, the rest being placed in the category of BR precursors (Zullo and Kohout, 2004). BRs containing 23-epoxy groups, 23-glycosidic groups, 23-ester groups, 3-oxygenated-5α-cholestane-23α diols along with alkyl derivatives were also considered natural BRs (Zullo, 2018). As reported, the bioactive potential of BRs lies with the side chains of rings (Zullo and Adam, 2002). Employment of the rice laminal inclination assay for ring structures showed that 22α and 23α dihydroxy BRs were active as 28-epibrassinolides (Watanabe et al., 2001). However, 23-dehydrogenation or conjugation of side chains may disrupt the biological activity (Watanabe et al., 2001). Further studies demonstrated that the biological activity of BRs was regulated by other active sites (Liu et al., 2017). A wide spectrum of structural variability was seen in ring A with fifteen types of structures starting from Δ2, 3-unsaturated to conjugated BRs. Even if analysis of the structural variation does not reveal the probable structures of this ring, it is assumed to belong to the category of BRs (Zullo, 2018). The biological functionality of BRs possessing rings increases in the order from 3β-hydroxy to 3α-dihydroxy rings (Liu et al., 2017). This variation in rings correlates with the biosynthetic pathway of BRs, suggesting that higher oxidation states are enhanced from the 6-deoxo and 6α-hydroxy forms toward the 6-oxo and 7-oxa lactone states (Vriet et al., 2013). It has been observed that there is decline in the functionality from 2α, 3α toward 2β, 3β and it is suggested that this decline is due to reason that α-oriented hydroxyl group that is present at C-2 is important for its activity (Zou et al., 2020). BRs have also been classified according to B-rings: 6-oxo-7-oxalactonic BRs (28-homobrassinolides, 2α,3β-dihydroxylated: 3-epibrassinolide, 2α,3α-dihydroxylated: brassinolide, 3β-hydroxylated: 3-epi-2-deoxybrassinolide, dolicholide), 6-oxo or 6-keto BRs (2α,3α-dihydroxylated: castasterone, 28-homocastasterone, 25-methylcastasterone, 2β,3α-dihydroxylated: 2-epicastasterone, 2α,3β-dihydroxylated: 3-epicastasterone, 2,3-diepi-25-methyl dolichosterone, 3β-monohydroxylated: teasterone, 28-homotyph asterol, 23-dehydro-2-epicastasterone, 1α,2α,3β-dihydroxylated: 1α-hydroxy-3-epicastasterone, 1α,2α,3β-dihydroxylated: 1α-hydroxy-3-epicastasterone, 23α-conjugates: 23-*O*-β-*D*-glucopy ranosyl-25-methyldolichosterone, 3-dehydro: 3-dehydrotea sterone), 6α-hydroxy BRs (6α-hydroxycastasterone), 6-deoxo BRs (2α,3α-dihydroxylated: 6-deoxocastasterone, 6-deoxo-28-homodolichosterone, 6-deoxo-24-epicastasterone, 3α-monohy droxylated: 6-deoxotyphasterol, 6-deoxo-28-homotyphasterol, 6-deoxo-28-norteasterone, 3-dehydro-6-deoxo-28-norteasterone), respectively (Zullo and Bajguz, 2019).

## Biosynthetic Pathways of BRs

Genetic and biochemical studies have identified most of the enzymes associated with BR synthesis up to now, but the molecular mechanism of BR biosynthesis and release needs further work to provide a deeper understanding of the regulation of its synthesis, catalysis and conjugation. These activities were explained in a coordinated manner by Japanese scientists who systematically elucidated the biosynthetic pathways concerning BRs within plant cells (Fujioka and Sakurai, 1997). Their data illustrated that biosynthesis of brassinolide (28-epibrassinolide) was linked to two main pathways, the early and late C6 oxidation pathways (Fujioka et al., 1998). The plant steroid, campesterol, was thought to be the progenitor of brassinolides on account of the side chain as well as the bioactive potential. The molecular structure and concurrence of teasterone, typhasterol, and castasterone also suggested that brassinolides were synthesized from campesterol (Yokota et al., 1991). The BR-specific campesterol precursor is initially transformed into campestanol, after which the early as well as late C6-pathways of oxidation, also termed as campestanol-dependent pathways, are activated. The enzymes required for this biosynthetic process include DET2, DWF4, cytochrome monooxygenases (Cyp P450), ROT3, BAS1, BR oxidase 2(BRox2), constitutive photomorphogenesis and dwarfism (CPD), and BR oxidase 6(BR6ox1) (Bhanu, 2019). The synthesis of castasterone and brassinolide requires the enzyme Cyp450 (CYP85A2), that also contributes to the rate limiting step for the conversion of 6-deoxycatasterone into castasterone and finally into brassinolide (Kim et al., 2005). Thus, by acting on several intermediates upstream, DWF4 can bifurcate the pathway at campesterol to generate an initial C-22 hydroxylation pathway (Fujioka and Yokota, 2003).

The BR biosynthetic pathway is generally triterpenoid in nature; therefore, the triterpene squalane can also be a precursor for BR synthesis, where it gets cyclized to cycloartenol using specialized enzymatic machinery (Hartmann, 1998). It is mainly formed via condensation of two molecules of FPP using NADPH as reducing agent (Heldt and Piechulla, 2011). In addition, plant sterols containing different alkyl groups at C24 like cholesterol and sitisterol, as well as campesterol, can also act as precursors for different BRs (Fujioka and Yokota, 2003). The biosynthetic pathway of BRs is limited at various steps through regulatory feedback processes to maintain endogenous BR homeostasis by the expression of genes for BR degradation. For example, expression of the *CPD*, *DET2*, and *DWARF4* genes is known to get modulated under such conditions. Also, those genes functioning in the C-22 hydroxylase pathway become active during the DWF4- or CPD-induced cascade depending on the availability of substrates. DWF4 can also bind large substrates like campesterol, 4-en-3-one, campestanol, and 6-oxo campesterol to enter the C-22 oxidation biosynthetic pathway. Likewise, CPD can metabolize campesterol and 22, 23-dihydroxy campesterol via parallel pathways. After campesterol hydroxylation by DWF4, the intermediates formed are altered and enter the 6-oxidation pathway. It was also assumed that *CYP90A1/CPD* encoding C23 hydroxylases converted teasterone into castasterone (Szekeres et al., 1996; Chung and Choe, 2013). *CYP90A1/CPD* was reported to be active in the C3 oxidation pathway as revealed through GC-MS analysis (Ohnishi et al., 2012). The transformation of 6-deoxo teasterone to 6-deoxo dehydroteasterone can also occur through CYP90A1/CPD activation. However, 22-hydroxycampesterol accumulation was observed along with reduced 6-deoxo 3-deoxyeasterone and 6-deoxo castasterone in CPD-knockout mutants. By this, it was inferred that the campestanol in-mediated pathway was principally involved in BR biosynthesis. CYP90C1/ROT3 and CYP90D1 were also important in C23 hydroxylase synthesis. This adds a new dimension to the BR synthesis pathway through conversion of 22-hydroxy-ergost-4-en-3-one and 22-hydroxy-5α-ergostan 3-one into C23 hydoxylated forms (Bishop, 2007). During the early stage of the C6 oxidation pathway, oxidation at C-6 occurs before the onset of DWF4-induced C22 hydroxylation. Studies were done with *Arabidopsis* CYP85A1 and CYP85A2 on the C-6 oxidation pathway that showed conversion of 6-deoxoteasterone and 6-deoxocastasterone into typhasterol and castasterone (Kim et al., 2005). CYP85A2 was also observed to induce the conversion of 6-deoxotyphasterol into brassinolides through castasterone by a downstream pathway. An investigation in CYP85A1 and CYP85A2 double mutants of endogenous BR levels suggested that increased levels of 6-oxocampesterol were present at the initial steps of BR-biosynthesis, revealing the activity of an unknown enzyme (Kwon et al., 2005). It is pertinent to mention here that CYP85A2-induced brassinolide synthesis only occurs in dicots. In rice, which is a monocot, brassinolides were not detected even in BRI1 mutants (Yamamuro et al., 2000). Also, monocots have only single copy *CYP85A* gene, while, dicots have duplicate genes (Kim et al., 2008). The evidence shows that castasterone is the product formed using these mechanistic pathways (Kim et al., 2008), but the additional genetic and biochemical strategies that enhance BR biosynthesis also need to be determined.

**FIGURE 1:**
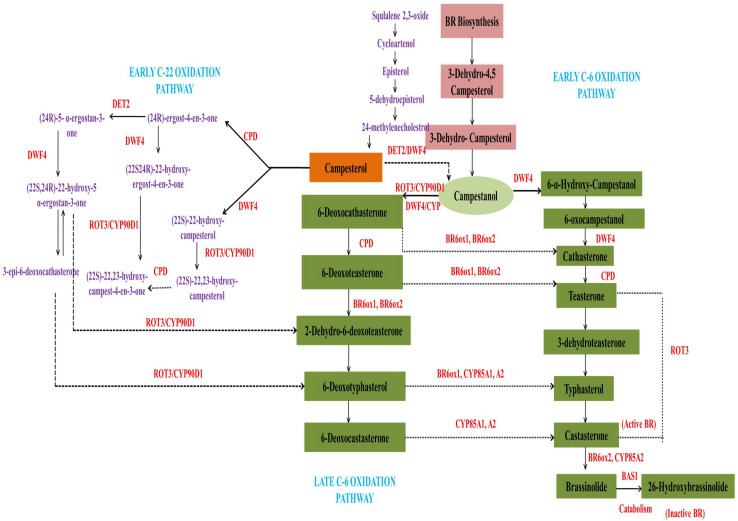
Schematic representation of the BR biosynthetic pathway, showing the early C-6 oxidation pathway, the late C-6 oxidation pathway and the early C-22 oxidation pathway.

The accumulation of BZR1 (brassinazole-resistant 1) and BRI1 EMS suppressor BES1/BZR2 reduced the expression of BR biosynthetic genes (Bartwal and Arora, 2020).*BAS1*, which encodes PhyB-activated tagged suppressor 1, is the foremost BR-inactivating gene in plants for maintaining optimal hormone levels. Mutations in the pathway for BR synthesis have also been implicated in male sterility, leaf curl, and dwarfing and BR-induced signal transduction is essential in plant metabolism (Bartwal and Arora, 2020). Earlier studies on mutants showed that exogenous application of BRs in wild type plants restored mutant BR-biosynthetic genes, while this did not occur in strains where BR signaling was hindered. Later studies were conducted on mutants of *Arabidopsis*, to elucidate the BR-signaling and transcriptional regulatory process. It was reported that leucine-rich repeated receptors and protein kinases (LRR-RLK) and BRI1 were the primary receptors for BRs. The resulting signal cascade led to trans-phosphorylation of BRI1 and BAK1, and further downstream signal transduction events (Cheng et al., 2017). Figure 1 represents the detailed biosynthetic pathway of BRs showing the early C-6 oxidation pathway, late C-6 oxidation pathway and early C-22 oxidation pathway.

## Regulation of BR Biosynthesis

### Transcriptional Regulation

With evolution, plants have developed strategies for optimal growth with minimal energy input. When BRs levels are adequate, the regulatory mechanism of positive and negative feedback loops are activated to down-regulate the endogenous BR levels. The endogenous BR level is either regulated by the down-regulation of BR biosynthetic genes or by the inactivation of already available bioactive BRs. Exogenous BL application down-regulates the BR biosynthetic genes, DWF-4, CPD, BR6ox1, and ROT-3 (rotundifolia 3), but up-regulates Brz, an inhibitor of BR synthesis. Exogenous BL also up-regulates BAS-1, another inhibitor of BR synthesis and down-regulates BES/BZR, a key transcription factor in the BR biosynthesis and signaling network (Yu et al., 2011).

DWF-4 genes expressed in bacterial cells were found to not be allosterically regulated. Exogenous application of epi-BR or Brz inhibitors of BR biosynthesis altered the functioning of DWF-4 genes and the transcription of DWF-4 exceeded the threshold levels, even in BR signaling mutants. The accumulation of DWF-4 mRNA was observed after Brz application, but BL reduced DWF-4 mRNA. Some other genes like *CPD, BR6ox1*, and *ROT-3* were transcriptionally regulated by BL and Brz, but *DET-2* was sensitive to Brz only (Tanaka et al., 2005). The sterol methyltransferase 2 (*SMT2*) and Dwarf1(*DWF1*) genes related to sterol biosynthesis are sensitive to both BL and Brz, but the sterol C-14 reductase, FACKEL (FK), is responsive to Brz only. In Bri1 mutants exposure to BL and Brz can trigger the expression of DWF-4 but the other genes required the normal BRI1 for transcriptional regulation (Tanaka et al., 2005).

**FIGURE 2:**
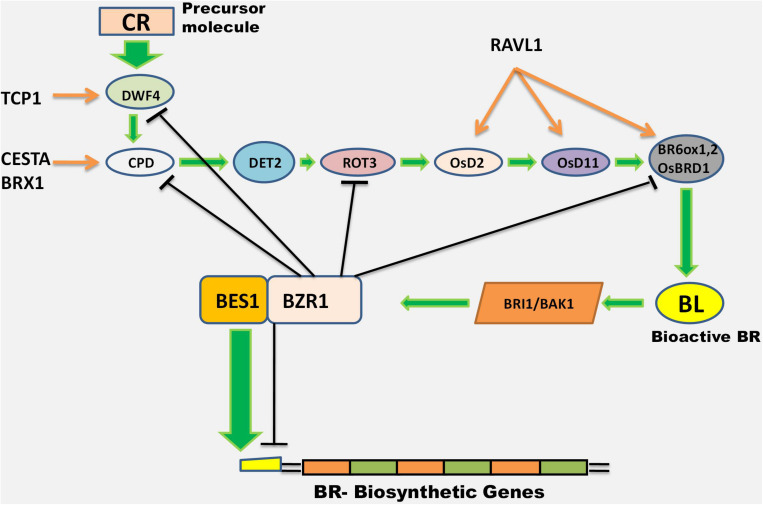
Outline of the transcriptional regulation of BR biosynthetic genes. BZR1 controls the expression of these genes through a negative feed-back loop (shown by black arrows). BES1, another downstream transcription factor, positively regulates the expression of BR-biosynthetic genes (shown as green and brown arrows) (It is a conclusion diagram based on reports in corresponding section “Transcriptional Regulation”).

Wang et al. (2002) reported that the two downstream factors BES and BZR controlled the whole BR regulation pathway. BZR1 is involved in transcriptional regulation of multiple BR-biosynthesis genes necessary for triggering specific growth processes and in down-regulating these genes by negative feed-back mechanism (He et al., 2005). At low BL levels, BIN2 phosphorylates BZR1, which inactivates it and blocks initiation of the BR signaling cascade. Normal BL levels initiate the BR signaling cascade, which either activates or inhibits the expression of intermediate genes. Thus, starting from campesterol as the primary precursor for BL formation, and proceeding through the activation or inactivation of BR-related genes, there exists a regulatory feed-back mechanism for controlling BR biosynthesis (Figure 2).

### Key BR Biosynthetic Enzymes and Their Transcriptional Regulation

#### Regulation of *CPD* (Constitutive Photo-Morphogenesis) and Dwarfism

In the brx^*s*^ mutant of *Arabidopsis*, expression of the *CPD* gene is arrested, which results in defective cell division and altered root growth (Mouchel et al., 2006). Normal BRX is required for *CPD* expression, as evidenced by exogenous BL application or by over-expression of CPD genes that convert the mutant root trait into the wild type one (Poppenberger et al., 2011). CESTA (CES), a basic helix-loop helix (bHLH) transcription factor, is a well-known trigger of CPD gene expression in *Arabidopsis*. CES binds to the CPD promoter region at the G-Box sequence elements and modulates its transcription.

#### Regulation of DWF4

Data from ChIP (chromatin immunoprecipitation) assays has shown that the expression of DWF4 can be regulated by TCP1 via direct or indirect binding to the DWF-4 promotor. TCP1 expression is up-regulated by BL treatment but down-regulated by the application of Brz (Guo et al., 2010).

#### D2, D11, and BRD1 Regulation

RAVL-1 and BRI-1 exhibit similar activity in loss of function and gain of function BR synthesis mutants. In-vivo and in-vitro experiments revealed that RAVL-1 bound to the B-box motif of the BR biosynthetic genes, *D2*, *D11*, *BRD-1*, and *OsBRI1*, and controlled their expression (Je et al., 2010).

### Auxin-Mediated BR Biosynthesis

In Arabidopsis, auxin can induce the expression of DWF4. Treatment with the auxin, indole acetic acid (IAA), or its synthetic analog, 2,4-D, up-regulates the expression of DWF4 and other intermediates such as 22-OHCR and 22-OH-3, and typically enhances endogenous BR levels in roots (Chung et al., 2011; Yoshimitsu et al., 2011). Thus, auxin signaling is required for independent DWF4 regulation with respect to BR biosynthesis. BZR1 binds strongly to the BRRE motif on the DWF-4 promotor with or without BL treatment. The DWF4 promotor was found to have multiple regulatory sequences including one Aux/IAA response element (Aux/IAA-RE, TGTCTC) (Ulmasov et al., 1997), three BRRE motifs (Chung et al., 2011) and one Aux/IAA-RE-like element (TGTGCTC) (Hagen and Guilfoyle, 2002). The auxin response factor, ARF-7, also directly bound to the DWF4 promotor. At high auxin concentrations, the inhibitor of Aux/IAA was degraded with the release of ARF-7, which then bound to the DWF-4 promotor and initiated the expression of BR response and BR biosynthetic genes. It was also found that BZR1 left its binding site to accommodate the binding of ARF-7. CHX treatment masked the expression of DWF-4, which suggests that some other transcription factor must be required to mediate auxin-dependent DWF-4 regulation. One study conducted at a higher temperature (29°C) supported the hypothesis that auxin is involved in the expression of DWF-4 (Maharjan and Choe, 2011). Seedlings of a DWF-4 proGUS line showed increased DWF-4 gene expression at the higher ambient temperature. Hypocotyl elongation is stimulated by auxin, and higher temperature increases the synthesis and transport of auxins (Gray et al., 1998). Hypocotyl elongation was arrested in BR-biosynthetic mutants, and it was concluded that high BR levels were required for hypocotyl elongation under temperature stress (Nemhauser et al., 2004).

### Role of BZR1 and Other Transcriptional Regulators

Brassinosteroids are recognized by the receptor kinase, BRASSINOSTEROID-INSENSITIVE 1 (BRI1), and the subsequent downstream signal cascade activates the brassinazole-resistant 1 (BZR1) family of transcription factors, which plays a vital role in brassinosteroid-regulated gene expression (Guo R. et al., 2013). When the levels of BRs are low, BZR1 is phosphorylated by the BRASSINOSTEROID-INSENSITIVE 2 (BIN2) GSK3-like kinase. As a result, its DNA-binding activity is lost and it remains in the cytoplasm. When BR levels are high, BZR1 is dephosphorylated by protein phosphatase 2A (PP2A). Activation of BRI1 results in a sequence of phosphorylation reactions which activates brassinosteroid-signaling kinases (BSKs), cytoplasmic kinase (CDG1) and members of the BRI1 Suppressor 1 (BSU1) family. This phosphatase activity leads to the inactivation of glycogen synthase kinase 3 (GSK3) through dephosphorylation of tyrosine. Dephosphorylated BZR1 is translocated into the nucleus where it regulates various target genes (Bai et al., 2012). It was documented in *Arabidopsis* that BRZ1 accumulated in the nucleus at elevated temperatures where it induced the expression of growth-promoting genes. BZR1 induced the expression of PIF4 by binding to its promoter and acting as a temperature-dependent positive growth regulator (Ibañez et al., 2018). A recent study revealed that organ size in *Arabidopsis* is regulated by BZR1. Enhanced expression of *Zea mays* BZR1 resulted in phenotypes of enlarged cotyledons, and increased size of floral organs, rosette leaves and seeds in transgenic *Arabidopsis* (Zhang et al., 2020).

BZR1 and BES1 have been documented as positive regulators of freezing tolerance. Gain-of-function mutants (bzr1-1D and bes1-D) of the transcription factors, BZR1 and BES1, showed increased tolerance to freezing, and the accumulation of dephosphorylated BZR1 upon cold treatment (Li et al., 2017). Chen et al., 2019 revealed that BZR1 was essential for locule development in *Arabidopsis*. Disruption of all *BZR*s resulted in generation of a hextuple mutant (*bzr-h*) which showed vegetative growth phenotypes that were similar to those of the BR receptor null mutant. Yin et al. (2018) demonstrated the role of BRZ1 in heat stress tolerance in tomato by regulating the feronia (FER) homologs. Mutations in BZR1 impeded the respiratory burst oxidase homolog 1 (*RBOH1*) generation of hydrogen peroxide (H_2_O_2_) in the apoplast and heat tolerance. Addition of exogenous H_2_O_2_ restored the heat tolerance of the tomato *bzr1* mutant, and enhanced expression of BZR1 increased the generation of apoplastic H_2_O_2_ and improved heat stress tolerance.

### Role of Biosynthetic Inhibitors

Brassinazole (Brz) was reported as the first brassinosteroid biosynthesis inhibitor. *Arabidopsis thaliana* treated with Brz showed the phenotype of a brassinosteroid mutant but application of brassinolide restored wild type activity (Nagata et al., 2000). The triazole compound, propiconazol (Pcz), is a potent BR metabolism inhibitor in plants. *Arabidopsis* seedlings treated with Pcz showed various BR-deficient phenotypes like reduced primary root growth, reduced size of cotyledons, and epinastic growth of cotyledons. However, seedlings co-treated with Pcz and 24-epibrassinolide showed improved root length compared to controls (Hartwig et al., 2012). YCZ-18 is another BR inhibitor that targets C23-hydroxylation in the BR biosynthetic pathway. Wild-type *Arabidopsis* plants grown in medium supplemented with YCZ-18 had short opened cotyledons. A decline in endogenous BR levels was also observed in *Arabidopsis* treated with YCZ-18 (Oh et al., 2015a). A pyrimidine-type fungicide, fenarimol, was also reported to inhibit BR biosynthesis in *Arabidopsis* and cause loss of etiolation and dwarfism in the dark (Oh et al., 2015b). An imidazole fungicide, Imazalil used as a post-harvest antifungal agent binds to CYP51 and inhibits fungal biosynthesis of ergosterol. In *A. thaliana*, hypocotyl shortening was observed with Imazalil treatment, which was reversed by application of 24-epibrassinolide (Rozhon et al., 2019). It has been reported by Bajguz, 2019 that brassinozole (BRz) decreases the level of BRs in the leaves of Barley and this effect is reversed by the application of BRs exogenously. Voriconazole, fenpropimorph, and fluconazole are also some of the BR inhibitors that inhibit the synthesis of cycloeucalenol-obtusifoliol isomers that is responsible for decreasing the synthesis of BRs (Rozhon et al., 2019).

## Advances in BR Signal Transduction

The metabolic pathways associated with BRs are related to successive signaling networks. Therefore, an inclusive understanding of BRs homeostasis in plants is imperative for establishing a comprehensive view of BR signaling cascades (Chung and Choe, 2013). The BR-linked pathways have been elucidated over the past decades and the studies revealed a complex BR signaling network with a crucial role in proper growth and development of plants (Anwar et al., 2018). The signaling cascade initiated by BR was categorized into three steps: (i) BR recognition and early activation of BRI1 receptor kinases, (ii) inactivation of BIN2 inhibitors, phosphatases, and kinases, and (iii) regulation of transcriptional factors such as BES1 and BZR1 (Clouse, 2011).

### Brassinosteroid Recognition and Early Activation of BRI1 Receptor Kinases

The BRs are initially recognized by brassinosteroid-insensitive 1 (BRI1) receptor kinase on the surface of the cells (Chakraborty et al., 2015). BRI1 is comprized of a leucine-enriched repeat receptor kinase with an extra-cellular domain that binds BRs and trans-phosphorylates signals. BRI1 is associated with a co-receptor, somatic embryogenesis receptor kinase (SERK), and belonging to a smaller LLRK family. BRI1 and SERK in combination form an active intricate complex that stimulates a downstream signal network involving activation of a wide array of kinases and phosphatases. Consequently, many transcription factors are activated which in turn alter the expression of specific genes (Belkhadir et al., 2012). Exogenously supplemented BRs bind to BRI1 and induce an association with BRI1-associated receptor kinase 1 (BAK1) and a disassociation of BRI1 kinase inhibitor 1 (BKI1). BKI1 is a membrane-bound negative regulator of BR signals, which combines with BRI1 and is responsible for prevention of co-receptor interference. It has been widely reported in *Oryza sativa* and *A. thaliana* plants (Jiang et al., 2015). Another protein, OsREM4.1, has similar functions to BRI1. It was identified in *O. sativa* and has corroborated that disruption of the BRI1/SERK complex suppresses downstream BR signaling. The expression of OsREM4.1 was positively modulated by ABA and elevated in response to increased ABA levels, and showed significant participation of the bZIP transcription factor (Gui et al., 2016). Various studies support the hypothesis that BRI1 is a multi-function kinase with the ability to phosphorylate Ser, Thr, and Tyr residues and it was affirmed that phosphorylation of Tyr residue is vital for specific aspects of BR signaling in plants (Oh et al., 2009). BRI1 was localized to the plasma membrane as a homodimer. It is ligand-independent and responsible for stabilization and initiation of BR binding and signaling (Albrecht et al., 2008). BAK1 (also termed SERK3) has been associated with phosphorylation of BRI1 and BAK1 both in-vivo and in-vitro (Wang et al., 2008). SERK4, also termed as BAK1-like (BKK1), has been shown to interact with BRI1 in-vivo in a BR-dependent manner (He et al., 2007). Another LRR RLK, FLS2, interacts with BAK1 and augments its function in plant defense (Bar et al., 2010). Karlova et al. (2006) observed a significant role for SERK1 in embryogenesis where it heterodimerizes with BRI1 and augments BR signaling.

In order to expound the entire mechanism of phosphorylation and oligomerization of BRI1 and BAK1 in response to exogenously supplemented BR, Wang et al. (2008) expressed various combinations of kinase-inactive and wild-type *Arabidopsis* BRI1 and BAK1 in the same transgenic plant. It was concluded that an active BRI1 was responsible for BR-dependent association of the pair, and not BAK1 kinase. Additionally, it was observed that when BAK1-green fluorescent protein (GFP) was highly expressed in the bri1-1 null mutant background, the phosphorylation was drastically lowered in BAK1-GFP. In-vitro studies indicated that BAK1 positively triggered BRI1 activity and that they can transphosphorylate each other at specific residues (Wang et al., 2008). The above studies with LC-MS/MS assays, biochemical analysis and functional characterization have aided in development of a new sequential transphosphorylation model of BRI1/BAK1 interaction. These studies further suggested that plant receptor kinases have certain properties similar to those of TGF-b and RTKs receptor kinases present in mammals, although they have plant-specific characteristics (Wang et al., 2008). Similar to BRI1, Tyr residue are also autophosphorylated and mutation studies revealed that Tyr phosphorylation regulated the expression of a BAKI subset in-vivo (Oh et al., 2010).

### Inactivation of BIN2 – Role of Inhibitors, Phosphatases, and Kinases

Increased BR signaling activity results in de-activation of brassinosteroidinsensitive-2(BIN2) kinase, which is considered the principal effect of BR signaling (Chung and Choe, 2013). Sequence analysis of BIN2 revealed significant similarity with mammalian glycogensynthasekinase3 (GSK3). There was also similarity to a member of a small family of ten related genes identified in *Arabidopsis* that have an essential role in BR signal transduction (Rozhon et al., 2010). The inactivation of BR results in activation of two related transcription factors, BRI-EMS suppressor-1(BES1) (Yin et al., 2005) and brassinazole-resistant 1 (BZR1) (He et al., 2005). BES1 is also known as BZR2 (Wang et al., 2002). More recent studies of downstream BR signaling in *Arabidopsis* have elucidated the association between BRI1 and BAK1 activation (Kim and Wang, 2010). Another observation by Wang and Chory (2006) using a yeast two-hybrid screen for BRI1 integrators suggested that BRI1 kinase inhibitor-1 (BKI1) acted as a negative modulator of BR signaling. In the absence of BAK1 it bound to BRI1 and inactivated its function and prevented further binding to BAK1.

Exogenous BR treatment results in dissociation of BKI1 from the outer cell surface and further inhibits the BR signaling network. Association of BRI1 and BKI1 was demonstrated in-vivo and in-vitro, which resulted in BKI1 phosphorylation and interaction with recombinant BRI1-CD in vitro. More recent observations showed that BKI1 interacted with BRI1 through a C-terminal 20-residue conserved segment (Jaillais et al., 2011). They additionally indicated that a peptide surrounding this binding site suppressed the association of BRI1 with BAK1. A Lys-Arg-enriched domain present within the BKI1 directed the protein toward the plasma membrane. This localization consequently phosphorylated Tyr-211 within the motif and released BKI1 from the plasma membrane in a BRI1- and BR-dependent manner. Over-expression of a BKI1-Y211F mutant construct in transgenic *Arabidopsis* consequently resulted in extremely dwarfed plants. This protein was reportedly membrane bound and found at the same location even after BR supplementation. This observation suggests that upon recognition, BR phosphorylates BRI1 on Tyr-211, resulting in its disassociation from the membrane. This permits BRI1 to freely associate with BAK1 and initiate BR signaling (Lemmon and Schlessinger, 2010; Lim and Pawson, 2010).

A proteomic screening of BR-modulated proteins identified a plethora of members of receptor-like cytoplasmic kinase families such as RLCK-XII, specifically BR-signaling kinases (BSKs). BSKs are a direct substrate of BRI1 and up-regulate BR signaling (Tang et al., 2008). BSKs have a presumed trans-membrane sequence with N-myristylation sites that are probably responsible for directing their membrane localization. Additionally, BSK1 and BSK3 have been observed to interact with BRI1 in-situ in the absence of other ligands. BRI1 phosphorylated BSK1, most likely on Ser-230, resulting in activation and its release from the receptor complex (Tang et al., 2008). This phosphorylated BSK1 interacted with BRI suppressor-1 (BSU1) phosphatase which then negatively modulated BIN2 (Kim et al., 2009). Ample biochemical and genetic evidence affirmed that BSK1 binding to BSU1 activated BSU1-mediated dephosphorylation of phosphoTyr-200 in BIN2, resulting in suppression of the BR signaling cascade (Kim et al., 2009).

### Regulation of Transcriptional Factors, BES1 and BZR

**FIGURE 3:**
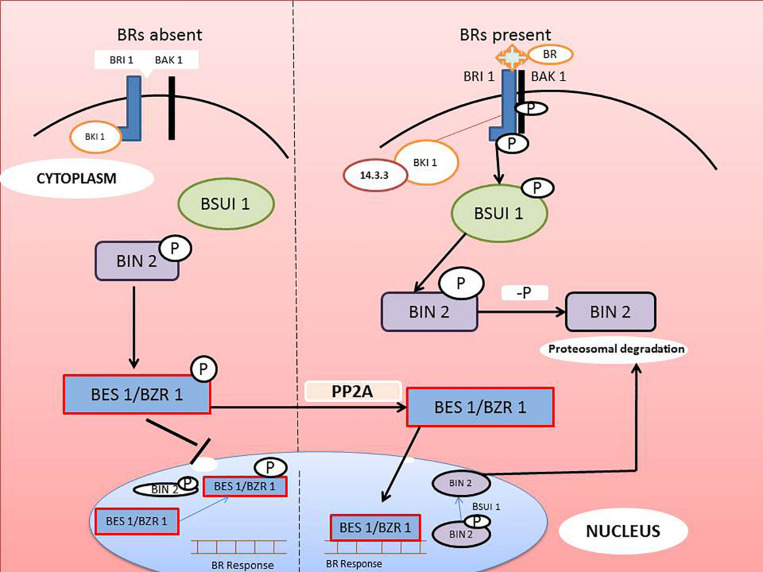
Molecular mechanism of BRs signaling from recognition at the cell surface to transcriptional activation of specific genes in the nucleus (modified from Chung and Choe, 2013).

Brassinazole-resistant 1(BZR1) and BRI 1-EMS suppressor-1 (BES1) are the chief transcriptional factors activated when BR binds to BRI1. BZR1 and BES1 (also known as BZR2) regulate a number of genes involved in various physiological processes (Peres et al., 2019). BIN2 (brassinosteroid insensitive-2), a GSK3/serine-threonine protein kinase, plays a key role in regulation of BZR1 AND BES1. In the BR-mediated signaling cascade, BIN2 acts as negative regulator by repressing the activity of BZR1 and BES1. In the absence of BR signaling, cytoplasmic CDG1 and BSKs interact with inactive BRI1 to prevent binding of BAK1 to the receptor. BES1 and BZR1 are phosphorylated by BIN2, and the phosphorylated transcription factors are expelled from the nucleus and retained in the cytoplasm by 14-3-3 proteins (Chung and Choe, 2013). The dephosphorylated BIN2 may be involved in degradation of BZR1 and BES1. Concomitantly, in the presence of BR, BRI1 recognized the signal followed by association with BAK1 and disassociation from BKI1. Auto- or trans-phosphorylation of BRI1 and BAK1 activates the main receptor, which is followed by phosphorylation of BSKs and CDG1. Activated BSKs and CDG1 stimulate BSU1 phosphatase, which dephosphorylates the major repressor protein, BIN2. Inactivation of BIN2 positively affects BZR1 and BES1 by terminating their degradation. In the absence of BIN2, phosphatase 2A dephosphorylates BZR1 and BES2 in the cytoplasm. The dephosphorylated transcription factors are than translocated to the nucleus where they affect expression of various BR-mediated genes. The mechanism of BRs signaling is illustrated in the Figure 3.

## Interaction of BES1 and BZR1 with Other Transcriptional Factors–Integration of Signaling Networks

In the BR-mediated signaling cascade, BES1 and BZR1 interact with the target genome product and play important roles in regulating gene expression. BES1/ BZR1 can act as an inducer as well as a repressor in the BR signaling pathway. BES1 binds to the CANNTG sequences (E-box) to stimulate gene expression, whereas BZR1 interacts with the CGTGT/CG sequences (BRRE) to suppress gene expression (He et al., 2005). MYB30, a BES1 target interacts with BES1 to strengthen BR induced signals. MYBL2 also functions as an important regulator in BR signaling. It is phosphorylated and stabilized by BIN2 kinase, thus playing an effective role in the transcriptional process of BR (Ye et al., 2012). Studies revealed that in the BR-repressed condition, there were more BRRE expressed, whereas E-boxes showed enhanced expression in BR-induced genes (Yu et al., 2011). The E-box interacts with BES1 and different transcription factors and cofactors. Interactions between BES1, PIL6, GLK1, and GLK2 have been associated with the transcriptional network pathway that triggers BR-induced gene expression. BZR1 interacted with PIF4 (a target gene product) to form a heterodimer that recognized a promoter element, CACGTG (G-box) (Oh et al., 2012). Interaction of BZR1 and PIFs are very important for normal growth of hypocotyls. Evidence has been obtained by studying mutants of PIF4 and the homologs (pif1 pif3 pif4 pif5), which showed reduced plant growth in darkness and inhibition of BR-induced growth. Plants have up to 2,000 target genes common for both BZR1 and PIF4, which also have various PREs involved in cell elongation (Guo H. et al., 2013). In the GA signaling pathway, in the absence of GA, the DELLA protein is stably localized to the nucleus and binds to PIFs and BZR, impeding the genome binding activity. An active GA signal leads to degradation of the DELLA protein thereby activating PIF and BZR, which target specific genes (Liu et al., 2018). Pioneer studies have revealed that in inhibition of chloroplast BES1 plays a role through GLK1 and GLK2. Inhibition of chloroplasts causes enlarged plastoglobules and other alterations in the structure of the organelle (Yu et al., 2011).

## Role of BRs in Plants Under Heavy Metal Stress

**FIGURE 4:**
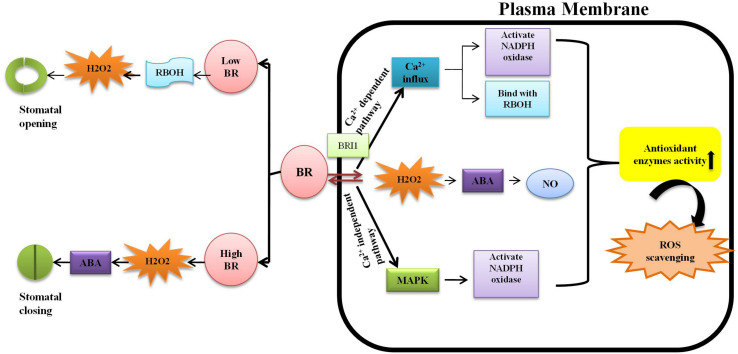
Different doses of Brassinosteroid regulates the opening and closing of stomata: at low concentration of BR activate respiratory burst oxidase homolog (RBOH) which further increases the level of H_2_O_2_ resulting in opening of stomata whereas at high concentration, it causes the accumulation of H_2_O_2_ and also reducing the uptake of K^+^ ions in guard cells lead to the activation of abscisic acid (ABA) cause closure of stomata (Daszkowska-Golec and Szarejko, 2013). Brassinosteroid hormone received by a specific receptor, brassinosteroid insensitive1 (BR1), on plasma membrane. Furthermore, there is increase in Ca^2+^ infux that activates the NADPH oxidase and then bind with EF-motif of RBOH enhancing the production of ROS (Xia et al., 2015); another pathway is upregulation of mitogen-activated protein kinase (MAPK) which further scavenge the reactive oxygen species (ROS); BR also stimulates the production of nitric oxide (NO) resulting the enhancement in antioxidative defense system (Zhang et al., 2011).

Plants survival is threatened in several ways but heavy metal stress is one of the most important concerns in agricultural research. The effects of metal contamination on plants is primarily ameliorated by hormones such as the BRs, that scavenge reactive oxygen species (ROS) and activate the antioxidant defense enzymes, superoxide dismutase (SOD), peroxidase (POD), catalase (CAT), ascorbate peroxidase (APOX), glutathione reductase (GR), guaiacol peroxidase (GPOX), and glutathione-S-transferase (GST) (Bücker-Neto et al., 2017). BRs help the plant to become metal-tolerant, thereby increasing crop yield and quality (Vriet et al., 2012). The bioactive BRs, 28-homobrassinolide (HBL) and 24-epibrassinolide (EBL), are part of a system called assisted phytoremediation, which helps the plant to eliminate toxic metals (Barbafieri and Tassi, 2011). BRs reduce the uptake of heavy metals by altering cell membrane permeability and also induce a group of defensive enzymes. They stimulate the production of stress-proteins through actions on anti-stress genes due to increased expression of ATPase (Madhan et al., 2014). External application of BRs induces transient H_2_O_2_ formation, which activates MAPK, leading to the production of NADPH oxidase (Jiang et al., 2012) and upregulation of stress-proteins and defensive enzymes that curtail the metal stress (Yin et al., 2016). Jiang et al. (2012) suggested that low doses of EBR (0.1–0.15 μM) suppressed photosynthetic efficiency. According to Xia et al. (2014), 0.1 μM EBR stimulated stomatal opening while 1.0 μM EBR facilitated the closing of stomata (Figure 4).

The mechanism of regulation of heavy metal toxicity via BRs involves: (a) stimulating H_2_O_2_ production, (b) scavenging ROS through boosting the defensive antioxidant system, (c) up-regulating MAPK expression, and (d) alleviating metal toxicity by increasing the concentration of potassium and sodium ions, proline, antioxidants, and osmolytes (Rajewska et al., 2016). Some reports indicated that NADPH oxidase was an important apoplastic source of H_2_O_2_ from conversion of O_2_^–^ by superoxide dismutase in the plasma membrane of plant cells, which further increased H^++^ ATPase activity via upregulation of the CsHA gene (Jakubowska et al., 2015). Thus, while uptake of toxic metals can negatively affect plant cell membranes by ROS peroxidation of lipids and oxidation of proteins, phytohormones like BRs boost the level of antioxidants and defensive enzymes to ameliorate this toxicity and restore normal osmoregulation (Shahzad et al., 2018). The BR-specific inhibitor, brassinazole (Brz), and the bioactive brassinosteroid, 24-brassinolide (EBL), were applied for alleviating the toxicity of metals, and restoring the photosynthetic machinery and defense system of *A. thaliana* (Wu et al., 2019). BRs are vital for plant cellular homeostasis. They restore CO_2_ absorption and enhance antioxidant capacity, thus overcoming the toxic effects of heavy metals (Ahammed et al., 2020). BRI1 is a BR receptor on the plasma membrane (Nolan et al., 2020) that activates the signaling cascade up-regulating the expression of transcription factors, which enhance the transcription of brassinosteroid genes (Tong and Chu, 2018; Planas-Riverola et al., 2019). Increased expression of these genes enhances the endogenous level of BRs that help in mitigating metal stress (Xia et al., 2018), but the mechanism remains unclear. Table 1 summarizes the role of brassinosteroids in regulation of physiological and biochemical responses of plants growing under metal toxicity.

**TABLE 1:** Some of the reports studied on role of brassinosteroids under heavy metal stress.

S.no.	Plant species	Metal concentration	Brassinosteroid concentration	Effect	References
1	*Brassica juncea*	150 mg/kg Mn	10^–8^ M EBL	Enhances ROS production, increases photosynthesis rate, restores stomatal opening and reduces electrolyte leakage	Hussain et al., 2019
2	*Glycine max* L.	20 μM Zn	100 nM EBL	Improves photosystem II; mitigates zinc stress by boosting antioxidant system and nutritional content; restores chloroplast membranes	dos Santos et al., 2020
3	*Vitis vinifera* L.	2 gL^–1^ Zn (ZnSO_4_⋅7H_2_O)	0.4 mg L^–1^ EBL	Increases photosynthetic rate and promotes grape productivity	Tadayon and Moafpourian, 2019
4	*Pisum sativum*	150 mg L^–1^ Cd	10^–7^ M EBL	Decreases methylglyoxal and hydrogen peroxide; alleviates electrolyte leakage; enhances glyoxylase I content and nutrient uptake by roots and shoots	Jan et al., 2018
5	*Brassica juncea* L.	2 mM Pb	10^–8^ M EBL	Eliminates Pb toxicity and increases protein content by reducing H_2_O_2_ and MDA	Dalyan et al., 2018
6	*Vitis vinifera* L.	120 μM Cu	0.10 mg L^–1^ EBL	Stimulates antioxidant system and alleviates oxidative damage by up-regulating activity of AsA-GSH cycle	Zhou et al., 2018
7	*Solanum lycopersicum*	3 and 9 mg kg^–1^ Cd	10^–8^ M HBL	Mitigates toxic effects of Cd on solanum seedlings by enhancing enzymes of photosystem II, carbohydrate and nitrogen assimilation	Singh and Prasad, 2017
8	*Arabidopsis thaliana*	50 μM Sb	10^–3^ M EBL	Mitigates toxic effects of Sb by activating antioxidant system	Wu et al., 2019
9	*Cucumis sativus*	10 μM Cd (CdCl_2_)	10 nM EBL	Enhances NADPH oxidase activity which causes accumulation of hydrogen peroxide and activation of the antioxidant system against Cd stress	Jakubowska and Janicka, 2017
10	*Solanum nigrum*	100 μM Ni(NiSO_4_⋅6H_2_O)	1 μM EBL	Raises Ni stress tolerance in *Solanum* by enhancing SOD activity; but, can cause down-regulation of APOX and CAT	Soares et al., 2016
11	*Oryza sativa* L.	0.5 mM Cr	0.1 nM EBL	Upregulates expression of CAT, APOX, and GR, thus increasing metal tolerance level in rice seedlings	Sharma P. et al., 2016
12	*Cajanus cajan* (L.) *Millsp.*	7.5 mM Al^3+^	0.5–2 μM EBL	Restores cellular homeostasis by reducing ROS and alleviating aluminum toxicity	Madhan et al., 2014
13	*Vigna radiata*	200 mg kg^–1^ Zn	10^–8^ M EBL	Improves plant growth by enhancing antioxidant activity	Mir et al., 2015
14	*Brassica juncea*	3 mM Mn	10^–8^ M EBL	Upregulates antioxidant defense system and photosynthetic efficiency of *Brassica* seedlings	Fariduddin et al., 2015
15	*Helianthus annuus*	80 μM Cu (CuSO_4_⋅5H_2_O)	100 μM EBL	Increases growth and metabolism of sunflowers	Filová et al., 2013
16	*Phaseolus vulgaris* L.	1 mM Cd^2+^ (CdCl_2_)	5 μM EBL	Increases activity of POD, GR, SOD, and CAT; down regulates MDA activity	Rady, 2011
17	*Raphanus sativus* L.	5 mM Zn(ZnSO_4_.7H_2_O)	2 μM HBL/EBL	Upregulates expression of SOD, APOX, POD, GR, and CAT; increases proline and chlorophyll content; restore nitrate reductase level in radish plants	Ramakrishna and Rao, 2015
18	*Brassica juncea*	0.75, 0.25, and 0.5 mM Cu	10^–7^ M and 10^–9^ M 24-EBL	Enhances antioxidant enzyme activity and reduces copper toxicity	Poonam et al., 2014
19	*Brassica juncea*	0.2 mM Cd	Endogenous	Upregulates 28-homobrassinolide in Brassica seedlings; enhances antioxidant enzyme production	Kapoor et al., 2014
20	*Vigna radiata*	100 or 150 mg kg^–1^ Ni(NiCl_2_)	10^–6^ M EBL	Activates peroxidase, catalase and superoxide dismutase; boosts proline content for nodulation and growth	Yusuf et al., 2012
21	*Raphanus sativus* L.	1.2 mM Cr(VI) (K_2_CrO_4_)	10^–9^ M EBL/1 mM spermidine	Stimulates production of plant hormones, IAA and ABA, and antioxidant enzymes, GR, SOD, CAT, and GPOX; increases content of proline, sugars, phytochelatins and pigments by decreasing MDA and H_2_O_2_	Choudhary et al., 2012a
22	*Raphanus sativus*	0.25 mM Cu (CuSO_4_⋅5H_2_O)	10^–9^ M EBR/1 mM spermidine	Increases seedling growth; boosts antioxidant system by up-regulating phytohormones, IAA and ABA	Choudhary et al., 2012b
23	*Solanum lycopersicum*	3–10 mg/kg Cd	10^–8^ M EBL/HBL	Increases antioxidant enzymes and photosynthetic pigments	Hayat et al., 2012
24	*Lycopersicon esculentum*	0.5-1.5 nM	10^–7^, 10^–9^,	Enhances Cd tolerance by up-regulating antioxidant system and protein content; decreases PPO and GST activity.	Sharma et al., 2014
		Cd+Hg	10^–11^ M HBL		
25	*Brassica juncea* L.	2 mM Ni	10^–9^ M EBL	Increases DHAR and GR activities of MDHAR and SOD enzymes	Kanwar et al., 2012
			10^–11^ M EBL		
26	*Lycopersicon esculentum*	3–12 mg/kg Cd	10^–8^ M EBL/HBL	Restores photosynthetic efficiency; boosts antioxidant defense response against Cd stress	Hasan et al., 2011
27	*Raphanus sativus* L.	1.0 mM Ni	10^–9^ M EBL	Mitigates metal-induced oxidative damage and activates antioxidant enzymes	Sharma et al., 2011
		1.0 mM Ni	10^–7^ M EBL		
		1.5 mM Ni	10^–9^ M EBL		
28	*Triticum aestivum*	50 and 100 μM Ni	0.01 μM HBL	Improves antioxidant system and upregulates CAT, POD, and SOD	Yusuf et al., 2011
29	*Lycopersicon esculentum*	100 or 200 μM	2 μM EBL	Increases expression of GSH, AsA and defense enzymes like SOD, GR, CAT, and APOX	Rady and Osman, 2012
		Pb^2+^ /Cd^2+^			
30	*Eucalyptus urophylla*	2.5 μM Fe (deficiency)	100 nM EBR	Increases consumption, transport and accumulation of iron (Fe) and other micronutrients in roots, leaves and stems	Lima et al., 2018
		250 μM Fe (control)			

## BRs Crosstalk with Other Phytohormones Under Heavy Metal Stress

**FIGURE 5:**
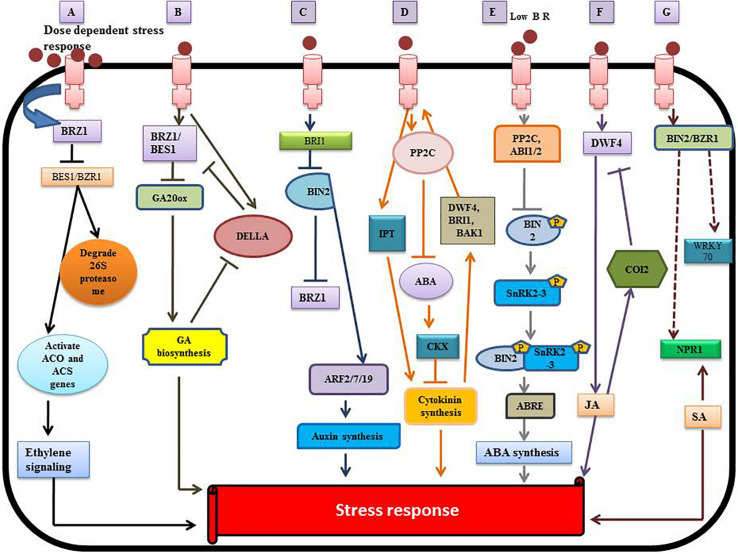
Crosstalk between BRs and other phytohormones. **(A)** Interplay between BR and ethylene. **(B)** Interaction between BR and gibberellin (GA). **(C)** Brassinosteroid – auxin crosstalk. **(D)** Brassinoster oid-cytokinin interaction. **(E)** Brassinosteroid-Abscisic acid (ABA) interplay. **(F)** Brassinosteroid-jasmonic acid (JA) crosstalk. **(G)** Brassinosteroid-salicylic acid (SA) [modified from Saini et al. (2015), Peres et al. (2019), and Ohri et al. (2019)], involving the regulation of various transcription factors.

Brassinosteroids play a diverse and vital role in regulating plant metabolism because of their synergy with other plant hormones such as auxin, cytokinins (CK), ethylene, polyamines (PA), gibberellins (GA), salicylic acid (SA), JA, and ABA (Ohri et al., 2019). The bioactive BRs (HBL, EBL) can protect the plant from toxic metals by assisted phytoremediation (Barbafieri and Tassi, 2011). BRs reduce the uptake of toxic metals by altering cell permeability and reduce damage by activating defensive enzymes. The mechanism of BRs signaling and its interplay with other hormones at the molecular level (Peres et al., 2019) is illustrated in Figure 5.

### Brassinosteroids and Ethylene

Brassinosteroids stimulate the synthesis of ethylene by activating the expression of ACO (1-aminocyclopropane-1-carboxylic acid oxidase) and ACS (1-aminocyclopropane-1-carboxylic acid (ACC)-synthase enzyme) genes (Figure 5). BR regulates ethylene synthesis at the transcriptional and post-transcriptional level by increasing the ACS5 protein half-life (Hansen et al., 2009). BR regulates ethylene biosynthesis negatively or positively via a dose-dependent pathway (Lv et al., 2018). Some reports showed that exogenous application of BR enhanced the ripening of banana (*Musa acuminata* L.) by elevating the expression of MaACS1, MaACO13, and MaACO14 genes and regulating ethylene production (Guo et al., 2019). BR acts at the post-transcriptional level by increasing the expression of ACS2 and ACS4 in *Solanum lycopersicum* (Zhu et al., 2015). BR also stabilizes other ACS proteins like ACS6, ACS9 and ACS5 by degrading the 26S proteasome (Hansen et al., 2009). BR indirectly regulates the hyponastic growth by regulating expression of the ROT3/CYP90C1 gene to increase ethylene synthesis (Polko et al., 2013). The overproduction of BR genes (BRI1 and DWARF) enhances the biosynthesis of ethylene in tomato (Li and He, 2016; Nie et al., 2017).

### Brassinosteroids and Gibberellins

The interplay between BRs (BZR1/BES1) and gibberellin (DELLA) genes is complex (Sun et al., 2010; Sun, 2011; Tong et al., 2014; Hu et al., 2017), reflecting interaction with both proteins and DNA (Li Q.F. et al., 2018; Li W. et al., 2018). The exogenous application of BR activates BZR1, which upregulates the expression of GA20ox genes and enhances GA production (Stewart Lilley et al., 2013). The DELLA gene has an inhibitory effect on the transcriptional activity of BZR1, but GA induces the degradation of DELLA (Bai et al., 2012). Therefore, both GA and BR affect expression of the target gene, BRZ1 (Figure 5). Thus, BES1/BRZ1 promotes production of GA, resulting in greater degradation of DELLA (Ross and Quittenden, 2016). There is direct interaction between DELLA and BZR1 that inhibits the binding of BZR1 to DNA. This suppresses the signal that is required for maintaining the etiolation of seedlings and elongation of cells (Li and He, 2013). The level of ROS-scavenging enzymes increases because of the interaction between DELLA protein and BRZ1 under heavy metal stress (Achard et al., 2008).

### Brassinosteroids and Auxins

Both BRs and auxins are considered “master regulators” with synergistic effects on plant growth and development (Chaiwanon and Wang, 2015). BIN2-mediated phosphorylation reduces the inhibitory effect of auxin response factors (ARF2) (Vert et al., 2008) that leads to the enhancement of ARF promoter activities and stimulates the expression of BR-regulated genes promoting auxin synthesis. The indole-3-acetic acid/auxin (IAA/AUX) genes are also involved in BR-regulated auxin production (Li Q.F. et al., 2018; Li W. et al., 2018). The expression of genes associated with auxin transport such as PIN3, PIN4, PIN7, and LAX are suppressed by the BR signaling pathway (Nemhauser et al., 2004). This crosstalk suggests an interplay between auxin and BR in heavy metal tolerance through the auxin transport genes. Wang et al., 2015 reported that the movement and local concentration of auxin was regulated by the expression of CYP79B2, the ABCB family, Yucca (YUC), the PIN family, the Gretchen Hagen (GH3) genes, and phosphoribosyl anthranilate transferase 1 (PAT1), in response to heavy metals (Hacham et al., 2012). The auxin secretion transporters, PIN7 and PIN4, that regulate the movement and distribution of auxin, are managed by BES1 (Paponov et al., 2005). In the root elongation zone, BIN2 stimulates the post-transcriptional modification of ARF, and BZR1 transcriptionally activates ARF expression and auxin-responsive genes (Tian et al., 2018). Both ARF and auxin-related genes were suppressed by BZR1 in the quiescent region of the root (Chaiwanon and Wang, 2015). It has been reported in *A. thaliana* that auxin treatment increases DWF4 expression, which stimulates production of BR through the auxin-induced Bravis radix (BRX) gene (Chung et al., 2011); there is feedback inhibition of DWF4 by BR. Several reports discussed the adversary aspect of auxin and BR in controlling BR synthesis genes and the DWF4 gene (Maharjan et al., 2011).

### Brassinosteroids and Cytokinins

Cytokinins (CKs) are adenine-derived compounds, which regulate the plant growth processes under abiotic stress (Perilli et al., 2010). CKs stimulate the defense mechanism that mitigates heavy metal toxicity and restores the photosynthetic apparatus. Piotrowska-Niczyporuk et al., 2012 reported the alleviation of Cd toxicity by enhancing photosynthesis efficiency and the level of primary metabolites. Two key enzymes regulate the biosynthesis of CKs: isopentenyltransferases (IPTs) which promote synthesis of CKs and CK oxidase/dehydrogenases (CKXs) which suppress genes involved in CK synthesis. Both enzymes target the responses mediated by BR (Werner and Schmülling, 2009). Exogenous application of BR leads to overexpression of CKX3 and ectopic expression of BRI1, which increases the leaf and root length. This suggests crosstalk between BR and CKs, which may be involved in enhancing crop yield (Yuldashev et al., 2012). Some reports showed that interaction between BR and CKs resulted in the accumulation of anthocyanins (Yuan et al., 2014). ABA is also involved in the interaction between BR and CKs, as it suppresses BR synthesis during metal toxicity (Zhang et al., 2009). Therefore, there are three hormones interacting in a complex way that remains unclear.

### Brassinosteroids and Abscisic Acid

Abscisic acid is known to be a “sensing element” for abiotic stress that protects the plant from various kinds of stress (Fujii and Zhu, 2009). The BR and ABA interplay depend upon the regulation of gene expression and modulation of protein activity. The complex formed by the merging of histone deacetylase19 (HDAC19), topless (TPL/TPR) and BRI1-EMS suppressor1 (BES1) affects the E-box promoter causing suppression of ABA insensitive 3 (ABI3) gene expression in the presence of BR. There is also repression of ABI3 and ABI5 gene expression through the interaction of the BZR1 transcription factor with the ABI5 G-Box promoter sequences. This reduces the stress response by down regulating the ABA-regulated gene expression. BIN2 promotes activation of SnRK2.3 that stimulates the stress response at low levels of BR. ABA-related gene expression is upregulated through phosphorylation of the ABI5 transcription factor by BIN2 (Wang et al., 2018). Some reports also showed auto-stimulation of downstream expression of ABA-related SnRK2s genes and kinase activity (Belin et al., 2006; Yunta et al., 2011).

### Brassinosteroids and Jasmonic Acid

CORONATINE INSENSITIVE 1 (COI1) is an F-box protein responsible for JA signaling and responses in *A. thaliana* (Yan et al., 2009; Peng et al., 2011) reported that blocking JA signaling caused an accumulation of anthocyanins in *Arabidopsis* under the influence of brassinazole. Upregulation and downregulation of JA transcript factors as well as signaling genes depended upon the relative concentration of BR in plants (Peng et al., 2011). The exogenous application of JA decreased expression of the BR signaling gene, OSBRI1 and the BR biosynthesis gene, OsDWF4, revealing the interplay between JA and BR in *O. sativa* (Nahar et al., 2013).

### Brassinosteroids and Salicylic Acid

Activation of systemic acquired resistance (SAR) is a putative role of SA during abiotic and biotic stress (Roychoudhury et al., 2016). Divi et al., 2010, studied the induction of BR-mediated stress tolerance by regulating BIN2 and BZR1. During the stress response, the OsWRKY45 transcription factor was expressed in the presence of SA (Huangfu et al., 2016). Some reports revealed that the metabolite composition was affected by the cumulative action of both SA and BR (24-epibrassinolide) leading to a decline in lead toxicity (Kohli et al., 2018). The interplay between BR and SA may be due to the NPR1 gene (non-expressor of pathogenesis-related genes 1) which stimulates expression of the SA-related genes involved in plant defense (Ohri et al., 2015). NPR1 also regulates the BR signaling genes, BIN2 and BZRI, which induces stress tolerance in plants (Divi et al., 2010).

### Brassinosteroids and Polyamines

Polyamines are associated with disease and the aging process. Previous studies reported that PAs did not affect the biosynthesis and signaling of BR (Anwar et al., 2015), butco-treatment with BR and PAs improved tolerance against copper stress. Choudhary et al. (2012b), reported enhanced Cu tolerance in radish through exogenous application of EBR and spermidine (Spd), which decreased the uptake of Cu and upregulated the expression of RsCOPT2 and RsCOPT1 genes. BR controls the spermidine level in plants, which further raises the concentration of putrescine as required for stress tolerance as well as diminishing cadaverine levels to counterbalance the oxidative burst (Takahashi and Kakehi, 2010). Pál et al., 2017, showed that in cadmium toxicity, Pas caused phytochelatin production in *O. sativa*. Recent reports suggest that the crosstalk between BR and PAs requires modulation of the expression of various enzymes associated with PA synthesis and its interplay with other plant hormones. As summarized in recent findings, this crosstalk between BRs and other phytohormones as well as their interaction showed both positive and antagonistic effects in response to stress.

## Conclusion and Future Perspectives

In this review, we have discussed the molecular mechanisms involved in the biosynthesis of the BRs, the roles of various transcriptional factors on gene expression, the interactions with auxin and other molecules, and the modulating effects of BES1/BZR1 in the biosynthesis of BRs. The molecular mechanism of BR signaling from its recognition on the plasma membrane to the transcriptional activation of specific genes in the nucleus has also been reviewed in detail. As a leading example of the importance of the BRs, we examined their role in heavy metal stress and their crosstalk with other phytohormones under stress conditions. But more work needs to be done for a detailed understanding of how BRs are transported out of cells and how they protect the plant during oxidative stress. There is a knowledge gap that needs attention to unravel the interactions between these phytohormones and various metabolites and transcription factors, which will add a new direction to the study of stress responses in plants.

## Author Contributions

AS, ML, and RB designed the outline and revised the initial draft. All authors were equally involved in writing of current version.

## Conflict of Interest

The authors declare that the research was conducted in the absence of any commercial or financial relationships that could be construed as a potential conflict of interest.

## Abbreviations

ABI1/2: abscisic acid-insensitive 1 and 2
ABRE: abscisic acid response elements
ACO: 1-aminocyclopropane-1-carboxylic acid (ACC)-oxidase
ACS: 1-aminocyclopropane-1-carboxylic acid (ACC)-synthase enzyme
APOX: ascorbate peroxidase
BIN2: brassinosteroid-insensitive 2
BZR1: brassinazole-resistant 1
BZR1/BES1: brassinazole-resistant 1/BRI1-EMS suppressor1
CAT: catalase
Cd: cadmium
CKX: CK oxidases/dehydrogenases
Cr: chromium
Cu: copper
DHAR: dehydroascorbate reductase
EBL: 24-epibrassinolide
GPOX: guaiacol peroxidase
GR: glutathione reductase
GSH: reduced glutathione
GST: glutathione-S-transferase
H_2_O_2_: hydrogen peroxide
HBL: 28-homobrassinolide
Hg: mercury
IPT: isopentenyltransferases
MDA: malondialdehyde
MDHAR: monodehydroascorbate reductase
Mn: manganese
Ni: nickel
NPR1: non-expressor of pathogenesis-related genes 1
Pb: lead
POD: peroxidase
PP2C: protein phosphatase 2C
PPO: polyphenol oxidase
ROS: reactive oxygen species
Sb: antimony
SOD: superoxide dismutase
Zn: zinc.

## References

[B1] Achard P. Renou J. P. Berthomé R. Harberd N. P. Genschik P. (2008). Plant DELLAs restrain growth and promote survival of adversity by reducing the levels of reactive oxygen species. *Curr. Biol.* 18 656–660. 10.1016/j.cub.2008.04.034 18450450

[B2] Ahammed G. J. Li X. Liu A. Chen S. (2020). Brassinosteroids in Plant Tolerance to Abiotic Stress. *J. Plant Growth Regul*. 2020 1–14.

[B3] Albrecht C. Russinova E. Kemmerling B. Kwaaitaal M. de Vries S. C. (2008). *Arabidopsis* SOMATIC EMBRYOGENESIS RECEPTOR KINASE proteins serve brassinosteroid-dependent and-independent signaling pathways. *Plant Physiol.* 148 611–619. 10.1104/pp.108.123216 18667726 PMC2528080

[B4] Ali B. (2019). *Brassinosteroids: The Promising Plant Growth Regulators in Horticulture. In Brassinosteroids: Plant Growth and Development.* Singapore: Springer, 349–365.

[B5] Anwar A. Liu Y. Dong R. Bai L. Yu X. Li Y. (2018). The physiological and molecular mechanism of brassinosteroid in response to stress: a review. *Biol. Res.* 51:46. 10.1186/s40659-018-0195-2 PMC6231256 30419959

[B6] Anwar R. Mattoo A. K. Handa A. K. (2015). *Polyamine interactions with plant hormones: crosstalk at several levels. In Polyamines.* Tokyo: Springer, 267–302.

[B7] Bai M. Y. Shang J. X. Oh E. Fan M. Bai Y. Zentella R. (2012). Brassinosteroid, gibberellin and phytochrome impinge on a common transcription module in *Arabidopsis*. *Nat. Cell Biol.* 14, 810–817. 10.1038/ncb2546 22820377 PMC3606816

[B8] Bajguz A. (2010). An enhancing effect of exogenous brassinolide on the growth and antioxidant activity in *Chlorella vulgaris* cultures under heavy metals stress. *Environ. Exp. Bot.* 68 175–179. 10.1016/j.envexpbot.2009.11.003

[B9] Bajguz A. Tretyn A. (2003). The chemical characteristic and distribution of brassinosteroids in plants. *Phytochem* 62 1027–1046. 10.1016/s0031-9422(02)00656-8 12591256

[B10] Bajguz A. (2019). *Brassinosteroids in microalgae: application for growth improvement and protection against abiotic stresses. In Brassinosteroids: Plant Growth and Development.* Singapore: Springer, 45–58.

[B11] Bajguz A. (2002). Brassinosteroids and lead as stimulators of phytochelatins synthesis in *Chlorella* vulgaris. *J. Plant Physiol.* 159 321–324. 10.1078/0176-1617-00654

[B12] Bar M. Sharfman M. Ron M. Avni A. (2010). BAK1 is required for the attenuation of ethylene-inducing xylanase (Eix)-induced defense responses by the decoy receptor LeEix1. *Plant J.* 63 791–800. 10.1111/j.1365-313x.2010.04282.x 20561260

[B13] Barbafieri M. Tassi E. (2011). *Brassinosteroids for phytoremediation application. In Brassinosteroids: a class of plant hormone.* Dordrecht: Springer, 403–437.

[B14] Bartwal A. Arora S. (2020). Brassinosteroids: Molecules with Myriad Roles. *Co-Evol. Sec. Metabol*. 2020 869–895. 10.1007/978-3-319-96397-6_18

[B15] Belin C. de Franco P. O. Bourbousse C. Chaignepain S. Schmitter J. M. Vavasseur A. (2006). Identification of features regulating OST1 kinase activity and OST1 function in guard cells. *Plant Physiol.* 141 1316–1327. 10.1104/pp.106.079327 16766677 PMC1533939

[B16] Belkhadir Y. Jaillais Y. Epple P. Balsemão-Pires E. Dangl J. L. Chory J. (2012). Brassinosteroids modulate the efficiency of plant immune responses to microbe-associated molecular patterns. *Proc. Natl. Acad. Sci.* 109 297–302. 10.1073/pnas.1112840108 22087001 PMC3252953

[B17] Bhanu A. N. (2019). Brassinosteroids: Relevance in Biological Activities of Plants and Agriculture. *J. Plant Sci. Res.* 35 1–15. 10.32381/jpsr.2019.35.01.1

[B18] Bishop G. J. (2007). Refining the plant steroid hormone biosynthesis pathway. *Trends Plant Sci.* 12 377–380. 10.1016/j.tplants.2007.07.001 17693126

[B19] Bücker-Neto L. Paiva A. L. S. Machado R. D. Arenhart R. A. Margis-Pinheiro M. (2017). Interactions between plant hormones and heavy metals responses. *Genet. Mol. Bio.* 40 373–386. 10.1590/1678-4685-gmb-2016-0087 28399194 PMC5452142

[B20] Caño-Delgado A. Yin Y. Yu C. Vafeados D. Mora-García S. Cheng J. C. (2004). BRL1 and BRL3 are novel brassinosteroid receptors that function in vascular differentiation in Arabidopsis. *Development* 131 5341–5351. 10.1242/dev.01403 15486337

[B21] Chaiwanon J. Wang Z. Y. (2015). Spatiotemporal brassinosteroid signaling and antagonism with auxin pattern stem cell dynamics in *Arabidopsis* roots. *Curr. Biol.* 25 1031–1042. 10.1016/j.cub.2015.02.046 25866388 PMC4415608

[B22] Chakraborty N. Sharma P. Kanyuka K. Pathak R. R. Choudhury D. Hooley R. (2015). G-protein α-subunit (GPA1) regulates stress, nitrate and phosphate response, flavonoid biosynthesis, fruit/seed development and substantially shares GCR1 regulation in *A. thaliana*. *Plant Mol. Biol.* 89 559–576. 10.1007/s11103-015-0374-2 26346778

[B23] Chen J. Fei K. Zhang W. Wang Z. Zhang J. Yang J. (2021). Brassinosteroids mediate the effect of high temperature during anthesis on the pistil activity of photo-thermosensitive genetic male-sterile rice lines. *Crop J.* 9, 109–119.

[B24] Chen L. G. Gao Z. Zhao Z. Liu X. Li Y. Zhang Y. (2019). BZR1 family transcription factors function redundantly and indispensably in BR signaling but exhibit BRI1-independent function in regulating anther development in *Arabidopsis*. *Mole. Plant* 12 1408–1415. 10.1016/j.molp.2019.06.006 31229643

[B25] Cheng X. Gou X. Yin H. Mysore K. S. Li J. Wen J. (2017). Functional characterisation of brassinosteroid receptor MtBRI1 in *Medicago truncatula*. *Sci. Rep.* 7 1–12. 28839160 10.1038/s41598-017-09297-9 PMC5570916

[B26] Chi C. Li X. Fang P. Xia X. Shi K. Zhou Y. (2020). Brassinosteroids act as a positive regulator of NBR1-dependent selective autophagy in response to chilling stress in tomato. *J. Exp. Bot.* 71, 1092–1106. 10.1093/jxb/erz466 31639824

[B27] Choudhary S. P. Kanwar M. Bhardwaj R. Yu J. Q. Tran L. S. P. (2012a). Chromium stress mitigation by polyamine-brassinosteroid application involves phytohormonal and physiological strategies in *Raphanus sativus* L. *PLoS One* 7:e33210. 10.1371/journal.pone.0033210 22479371 PMC3315560

[B28] Choudhary S. P. Oral H. V. Bhardwaj R. Yu J. Q. Tran L. S. P. (2012b). Interaction of brassinosteroids and polyamines enhances copper stress tolerance in *Raphanus sativus*. *J. Exp. Bot*. 63 5659–5675. 10.1093/jxb/ers219 22915739 PMC3444278

[B29] Chung Y. Choe S. (2013). The regulation of brassinosteroid biosynthesis in *Arabidopsis*. *Crit. Rev. Plant Sci.* 32 396–410. 10.1080/07352689.2013.797856

[B30] Chung Y. Maharjan P. M. Lee O. Fujioka S. Jang S. Kim B. (2011). Auxin stimulates DWARF4 expression and brassinosteroid biosynthesis in *Arabidopsis*. *Plant J.* 66 564–578. 10.1111/j.1365-313x.2011.04513.x 21284753

[B31] Clouse S. D. (2011). Brassinosteroid signal transduction: from receptor kinase activation to transcriptional networks regulating plant development. *Plant Cell.* 23 1219–1230. 10.1105/tpc.111.084475 21505068 PMC3101532

[B32] Clouse S. D. Sasse J. M. (1998). Brassinosteroids: essential regulators of plant growth and development. *Annu. Rev. Plant Biol.* 49 427–451. 10.1146/annurev.arplant.49.1.427 15012241

[B33] Dalyan E. Yüzbaşıoğlu E. Akpınar I. (2018). Effect of 24-epibrassinolide on antioxidative defence system against lead-induced oxidative stress in the roots of *Brassica juncea* L. seedlings. *Russian J. Plant Physiol*. 65 570–578. 10.1134/s1021443718040118

[B34] Daszkowska-Golec A. Szarejko I. (2013). Open or close the gate–stomata action under the control of phytohormones in drought stress conditions. *Front. Plant Sci*. 4:138. 10.3389/fpls.2013.00138 PMC3652521 23717320

[B35] Divi U. K. Rahman T. Krishna P. (2010). Brassinosteroid-mediated stress tolerance in Arabidopsis shows interactions with abscisic acid, ethylene and salicylic acid pathways. *BMC Plant Bio.* 10:151. 10.1186/1471-2229-10-151 20642851 PMC3095295

[B36] dos Santos L. R. da Silva B. R. S. Pedron T. Batista B. L. da Silva Lobato A. K. (2020). 24-Epibrassinolide improves root anatomy and antioxidant enzymes in soybean plants subjected to zinc stress. *J. Soil Sci. Plant Nutri*. 20 105–124.

[B37] Fariduddin Q. Ahmed M. Mir B. A. Yusuf M. Khan T. A. (2015). 24-Epibrassinolide mitigates the adverse effects of manganese induced toxicity through improved antioxidant system and photosynthetic attributes in Brassica juncea. *Env. Sci. and Pollut. Res*. 22 11349–11359. 10.1007/s11356-015-4339-4 25804660

[B38] Filová A. Sytar O. Krivosudská E. (2013). Effects of brassinosteroid on the induction of physiological changes in *Helianthus annuus* L. under copper stress. *Acta Uni. Agri. et Silvi. Mend. Brun*. 61 623–629. 10.11118/actaun201361030623

[B39] Fujii H. Zhu J. K. (2009). Arabidopsis mutant deficient in 3 abscisic acid-activated protein kinases reveals critical roles in growth, reproduction, and stress. *Proc. Natl. Acad. Sci.* 106 8380–8385. 10.1073/pnas.0903144106 19420218 PMC2688869

[B40] Fujioka S. Sakurai A. (1997). Brassinosteroids. *Nat. Prod. Rep.* 14 1–10. 10.1007/978-94-017-0948-4_1 9121728

[B41] Fujioka S. Yokota T. (2003). Biosynthesis and metabolism of brassinosteroids. *Ann. Rev. Plant Biol.* 54 137–164. 14502988 10.1146/annurev.arplant.54.031902.134921

[B42] Fujioka S. Noguchi T. Yokota T. Takatsuto S. Yoshida S. (1998). Brassinosteroids in *Arabidopsis thaliana*. *Phytochem.* 48 595–599. 10.1016/s0031-9422(98)00065-x 9664702

[B43] Gray W. M. Östin A. Sandberg G. Romano C. P. Estelle M. (1998). High temperature promotes auxin-mediated hypocotyl elongation in Arabidopsis. *Proc. Natl. Acad. Sci.* 95 7197–7202. 10.1073/pnas.95.12.7197 9618562 PMC22781

[B44] Gui J. Zheng S. Liu C. Shen J. Li J. Li L. (2016). OsREM4. 1 interacts with OsSERK1 to coordinate the interlinking between abscisic acid and brassinosteroid signaling in rice. *Dev. Cell.* 38 201–213. 10.1016/j.devcel.2016.06.011 27424498

[B45] Guo H. Li L. Aluru M. Aluru S. Yin Y. (2013). Mechanisms and networks for brassinosteroid regulated gene expression. *Curr. Opin. Plant Bio*. 16 545–553. 10.1016/j.pbi.2013.08.002 23993372

[B46] Guo R. Qian H. Shen W. Liu L. Zhang M. Cai C. (2013). BZR1 and BES1 participate in regulation of glucosinolate biosynthesis by brassinosteroids in *Arabidopsis*. *J. Exp. Bot*. 64 2401–2412. 10.1093/jxb/ert094 23580754 PMC3654425

[B47] Guo Y. F. Shan W. Liang S. M. Wu C. J. Wei W. Chen J. Y. (2019). MaBZR1/2 act as transcriptional repressors of ethylene biosynthetic genes in banana fruit. *Physio. Planta.* 165 555–568. 10.1111/ppl.12750 29704245

[B48] Guo Z. Fujioka S. Blancaflor E. B. Miao S. Gou X. Li J. (2010). TCP1 modulates brassinosteroid biosynthesis by regulating the expression of the key biosynthetic gene DWARF4 in *Arabidopsis thaliana*. *Plant Cell*. 22 1161–1173. 10.1105/tpc.109.069203 20435901 PMC2879762

[B49] Hacham Y. Sela A. Friedlander L. Savaldi-Goldstein S. (2012). BRI1 activity in the root meristem involves post-transcriptional regulation of PIN auxin efflux carriers. *Plant Sign. Behav*. 7 68–70. 10.4161/psb.7.1.18657 22231282 PMC3357372

[B50] Hagen G. Guilfoyle T. (2002). Auxin-responsive gene expression: genes, promoters and regulatory factors. *Plant Mol. Bio*. 49 373–385. 10.1007/978-94-010-0377-3_9 12036261

[B51] Hansen M. Chae H. S. Kieber J. J. (2009). Regulation of ACS protein stability by cytokinin and brassinosteroid. *Plant J.* 57 606–614. 10.1111/j.1365-313x.2008.03711.x 18980656 PMC2807401

[B52] Hartmann M. A. (1998). Plant sterols and the membrane environment. *Trends Plant Sci.* 3 170–175. 10.1016/s1360-1385(98)01233-3

[B53] Hartwig T. Corvalan C. Best N. B. Budka J. S. Zhu J.-Y. (2012). Propiconazole Is a Specific and Accessible Brassinosteroid (BR) Biosynthesis Inhibitor for Arabidopsis and Maize. *PLoS One.* 7:e36625. 10.1371/journal.pone.0036625 22590578 PMC3348881

[B54] Hasan S. A. Hayat S. Ahmad A. (2011). Brassinosteroids protect photosynthetic machinery against the cadmium induced oxidative stress in two tomato cultivars. *Chemosphere* 84 1446–1451. 10.1016/j.chemosphere.2011.04.047 21565386

[B55] Hayat S. Alyemeni M. N. Hasan S. A. (2012). Foliar spray of brassinosteroid enhances yield and quality of *Solanum lycopersicum* under cadmium stress. *Saudi J. Bio. Sci.* 19 325–335. 10.1016/j.sjbs.2012.03.005 23961193 PMC3730949

[B56] He J. X. Gendron J. M. Sun Y. Gampala S. S. Gendron N. Sun C. Q. (2005). BZR1 is a transcriptional repressor with dual roles in brassinosteroid homeostasis and growth responses. *Science* 307 1634–1638. 10.1126/science.1107580 15681342 PMC2925132

[B57] He K. Gou X. Yuan T. Lin H. Asami T. Yoshida S. (2007). BAK1 and BKK1 regulate brassinosteroid dependent growth and brassinosteroid-independent cell-death pathways. *Curr. Biol.* 17 1109–1115. 10.1016/j.cub.2007.05.036 17600708

[B58] Heldt H. W. Piechulla B. (2011). *In the photorespiratory pathway phosphoglycolate formed by the oxygenase activity of Rubisco is recycled. Plant Biochem.* London: Academic Press, 193–209.

[B59] Hu S. Wang C. Sanchez D. L. Lipka A. E. Liu P. Yin Y. (2017). Gibberellins promote brassinosteroids action and both increase heterosis for plant height in maize (Zea mays L.). *Front. Plant Sci.* 8:1039. 10.3389/fpls.2017.01039 PMC5477294 28676808

[B60] Huangfu J. Li J. Li R. Ye M. Kuai P. Zhang T. (2016). The transcription factor OsWRKY45 negatively modulates the resistance of rice to the brown planthopper *Nilaparvata lugens*. *Int. J. Mol. Sci*. 17:697. 10.3390/ijms17060697 27258255 PMC4926322

[B61] Hussain A. Nazir F. Fariduddin Q. (2019). 24-epibrassinolide and spermidine alleviate Mn stress via the modulation of root morphology, stomatal behavior, photosynthetic attributes and antioxidant defense in *Brassica juncea*. *Physio. Mol. Bio. Plants*. 25 905–919. 10.1007/s12298-019-00672-6 31404216 PMC6656853

[B62] Ibañez C. Delker C. Martinez C. Bürstenbinder K. Janitza P. Lippmann R. (2018). Brassinosteroids dominate hormonal regulation of plant thermo morphogenesis via BZR1. *Curr. Biol.* 28 303–310. 10.1016/j.cub.2017.11.077 29337075

[B63] Jaillais Y. Hothorn M. Belkhadir Y. Dabi T. Nimchuk Z. L. Meyerowitz E. M. (2011). Tyrosine phosphorylation controls brassinosteroid receptor activation by triggering membrane release of its kinase inhibitor. *Genes Dev.* 25 232–237. 10.1101/gad.2001911 21289069 PMC3034898

[B64] Jakubowska D. Janicka M. (2017). The role of brassinosteroids in the regulation of the plasma membrane H+-ATPase and NADPH oxidase under cadmium stress. *Plant Sci.* 264 37–47. 10.1016/j.plantsci.2017.08.007 28969801

[B65] Jakubowska D. Janicka-Russak M. Kabała K. Migocka M. Reda M. (2015). Modification of plasma membrane NADPH oxidase activity in cucumber seedling roots in response to cadmium stress. *Plant Sci.* 234 50–59. 10.1016/j.plantsci.2015.02.005 25804809

[B66] Jan S. Alyemeni M. N. Wijaya L. Alam P. Siddique K. H. Ahmad P. (2018). Interactive effect of 24-epibrassinolide and silicon alleviates cadmium stress via the modulation of antioxidant defense and glyoxalase systems and macronutrient content in *Pisum sativum L*. seedlings. *BMC Plant Biol.* 18, 1–18. 10.1186/s12870-018-1359-5 30012086 PMC6048797

[B67] Je B. I. Piao H. L. Park S. J. Park S. H. Kim C. M. Xuan Y. H. (2010). RAV-Like1 maintains brassinosteroid homeostasis via the coordinated activation of BRI1 and biosynthetic genes in rice. *Plant Cell*. 22 1777–1791. 10.1105/tpc.109.069575 20581303 PMC2910978

[B68] Jiang J. Zhang C. Wang X. (2015). A recently evolved isoform of the transcription factor BES1 promotes brassinosteroid signaling and development in *Arabidopsis thaliana*. *Plant Cell.* 27 361–374. 10.1105/tpc.114.133678 25649439 PMC4456931

[B69] Jiang Y. P. Cheng F. Zhou Y. H. Xia X. J. Mao W. H. Shi K. (2012). Cellular glutathione redox homeostasis plays an important role in the brassinosteroid−induced increase in CO2 assimilation in Cucumis sativus. *New Phytol.* 194 932–943. 10.1111/j.1469-8137.2012.04111.x 22432590

[B70] Kang Y. Y. Guo S. R. (2011). *Role of brassinosteroids on horticultural crops. In Brassinosteroids: A class of plant hormone.* Dordrecht: Springer, 269–288.

[B71] Kanwar M. K. Bhardwaj R. Arora P. Chowdhary S. P. Sharma P. Kumar S. (2012). Plant steroid hormones produced under Ni stress are involved in the regulation of metal uptake and oxidative stress in *Brassica juncea* L. *Chemosphere* 86 41–49. 10.1016/j.chemosphere.2011.08.048 21959144

[B72] Kapoor D. Kaur S. Bhardwaj R. (2014). Physiological and biochemical changes in *Brassica juncea* plants under Cd-induced stress. *BioMed Res. Int*. 2:2014. 10.1155/2014/726070 PMC4123575 25133178

[B73] Karlova R. Boeren S. Russinova E. Aker J. Vervoort J. de Vries S. (2006). The Arabidopsis Somatic Embryogenesis Receptor-Like Kinase1 Protein Complex Includes Brassinosteroid- Insensitive1. *Plant Cell.* 18 626–638. 10.1105/tpc.105.039412 16473966 PMC1383638

[B74] Kim B. K. Fujioka S. Takatsuto S. Tsujimoto M. Choe S. (2008). Castasterone is a likely end product of brassinosteroid biosynthetic pathway in rice. *Biochem. Biophysio. Res. Commun.* 374 614–619. 10.1016/j.bbrc.2008.07.073 18656444

[B75] Kim T. W. Wang Z. Y. (2010). Brassinosteroid signal transduction from receptor kinases to transcription factors. *Annu. Rev. Plant Biol.* 61 681–704. 10.1146/annurev.arplant.043008.092057 20192752

[B76] Kim T. W. Guan S. Sun Y. Deng Z. Tang W. Shang J. X. (2009). Brassinosteroid signaltransduction from cell-surface receptor kinases to nuclear transcription factors. *Nat. Cell Biol.* 11 1254–1260. 10.1038/ncb1970 19734888 PMC2910619

[B77] Kim T. W. Hwang J. Y. Kim Y. S. Joo S. H. Chang S. C. Lee J. S. (2005). *Arabidopsis* CYP85A2, a cytochrome P450, mediates the Baeyer-Villiger oxidation of castasterone to brassinolide in brassinosteroid biosynthesis. *Plant Cell*. 17 2397–2412. 10.1105/tpc.105.033738 16024588 PMC1182497

[B78] Kohli S. K. Handa N. Sharma A. Gautam V. Arora S. Bhardwaj R. (2018). Combined effect of 24-epibrassinolide and salicylic acid mitigates lead (Pb) toxicity by modulating various metabolites in Brassica juncea L. seedlings. *Protoplasma* 255 11–24. 10.1007/s00709-017-1124-x 28573335

[B79] Kwon M. Fujioka S. Jeon J. H. Kim H. B. Takatsuto S. Yoshida S. (2005). A double mutant for theCYP85A1 andCYP85A2 Genes of *Arabidopsis* exhibits a Brassinosteroid dwarf phenotype. *J. Plant Biol.* 48 237–244. 10.1007/bf03030413

[B80] Lemmon M. A. Schlessinger J. (2010). Cell signaling by receptor tyrosine kinases. *Cell* 141 1117–1134. 10.1016/j.cell.2010.06.011 20602996 PMC2914105

[B81] Li Q.-F. He J.-X. (2013). Mechanisms of signaling crosstalk between brassinosteroids and gibberellins. *Plant Signal Behav.* 8:e24686. 10.4161/psb.24686 23603943 PMC3909037

[B82] Li H. Ye K. Shi Y. Cheng J. Zhang X. Yang S. (2017). BZR1 positively regulates freezing tolerance via CBF-dependent and CBF-independent pathways in *Arabidopsis*. *Mol. Plant*. 10 545–559. 10.1016/j.molp.2017.01.004 28089951

[B83] Li Q. F. He J. X. (2016). BZR1 interacts with HY5 to mediate brassinosteroid-and light-regulated cotyledon opening in *Arabidopsis* in darkness. *Mol. Plant*. 9 113–125. 10.1016/j.molp.2015.08.014 26363272

[B84] Li Q. F. Lu J. Yu J. W. Zhang C. Q. He J. X. Liu Q. Q. (2018). The brassinosteroid-regulated transcription factors BZR1/BES1 function as a coordinator in multisignal-regulated plant growth. *Biochim. Biophys. Acta* 1861 561–571. 10.1016/j.bbagrm.2018.04.003 29673687

[B85] Li W. Nishiyama R. Watanabe Y. Van Ha C. Kojima M. An P. (2018). Effects of overproduced ethylene on the contents of other phytohormones and expression of their key biosynthetic genes. *Plant Physio. Biochem*. 128 170–177. 10.1016/j.plaphy.2018.05.013 29783182

[B86] Lim W. A. Pawson T. (2010). Phosphotyrosine signaling: Evolving a new cellular communication system. *Cell* 142 661–667. 10.1016/j.cell.2010.08.023 20813250 PMC2950826

[B87] Lima M. D. R. Junior U. D. O. B. Batista B. L. da Silva Lobato A. K. (2018). Brassinosteroids mitigate iron deficiency improving nutritional status and photochemical efficiency in *Eucalyptus urophylla* plants. *Trees* 32 1681–1694. 10.1007/s00468-018-1743-7

[B88] Liu F. Wang P. Zhang X. Li X. Yan X. Fu D. (2018). The genetic and molecular basis of crop height based on a rice model. *Planta* 247 1–26. 10.1007/s00425-017-2798-1 29110072

[B89] Liu J. Zhang D. Sun X. Ding T. Lei B. Zhang C. (2017). Structure-activity relationship of brassinosteroids and their agricultural practical usages. *Steroids* 124 1–17. 10.1016/j.steroids.2017.05.005 28502860

[B90] Lv B. Tian H. Zhang F. Liu J. Lu S. Bai M. (2018). Brassinosteroids regulate root growth by controlling reactive oxygen species homeostasis and dual effect on ethylene synthesis in *Arabidopsis*. *PLoS Genetics* 14:e1007144. 10.1371/journal.pgen.1007144 29324765 PMC5783399

[B91] Madhan M. Mahesh K. Rao S. S. (2014). Effect of 24-epibrassinolide on aluminium stress induced inhibition of seed germination and seedling growth of *Cajanus cajan* (L.) *Millsp*. *Int. J. Multidiscipl. Curr. Res.* 2 286–290.

[B92] Maharjan P. M. Choe S. (2011). High temperature stimulates DWARF4 (DWF4) expression to increase hypocotyl elongation in *Arabidopsis*. *J. Plant Bio*. 54:425. 10.1007/s12374-011-9183-6

[B93] Maharjan P. M. Schulz B. Choe S. (2011). BIN2/DWF12 antagonistically transduces brassinosteroid and auxin signals in the roots of *Arabidopsis*. *J. Plant Bio*. 54 126–134. 10.1007/s12374-010-9138-3

[B94] Mir B. A. Khan T. A. Fariduddin Q. (2015). 24-epibrassinolide and spermidine modulate photosynthesis and antioxidant systems in Vigna radiata under salt and zinc stress. *Int. J. Adv. Res*. 3 592–608.

[B95] Mouchel C. F. Osmont K. S. Hardtke C. S. (2006). BRX mediates feedback between brassinosteroid levels and auxin signalling in root growth. *Nature* 443 458–461. 10.1038/nature05130 17006513

[B96] Nagata N. Min Y. K. Nakano T. Asami T. Yoshida S. (2000). Treatment of dark-grown Arabidopsis thaliana with a brassinosteroid-biosynthesis inhibitor, brassinazole, induces some characteristics of light-grown plants. *Planta* 211 781–790. 10.1007/s004250000351 11144262

[B97] Nahar K. Kyndt T. Hause B. Höfte M. Gheysen G. (2013). Brassinosteroids suppress rice defense against root-knot nematodes through antagonism with the jasmonate pathway. *Mol. Plant-Microbe Inter*. 26 106–115. 10.1094/mpmi-05-12-0108-fi 23194343

[B98] Nemhauser J. L. Mockler T. C. Chory J. (2004). Interdependency of brassinosteroid and auxin signaling in *Arabidopsis*. *PLoS Biol*. 2:e258. 10.1371/journal.pbio.0020258 15328536 PMC509407

[B99] Nie S. Huang S. Wang S. Cheng D. Liu J. Lv S. (2017). Enhancing brassinosteroid signaling via overexpression of tomato (Solanum lycopersicum) SlBRI1 improves major agronomic traits. *Front. Plant Sci*. 8:1386. 10.3389/fpls.2017.01386 PMC5554372 28848587

[B100] Nolan T. M. Vukašinoviæ N. Liu D. Russinova E. Yin Y. (2020). Brassinosteroids: Multidimensional regulators of plant growth, development, and stress responses. *Plant Cell*. 32 295–318. 10.1105/tpc.19.00335 31776234 PMC7008487

[B101] Nolan T. Chen J. Yin Y. (2017). Cross-talk of Brassinosteroid signaling in controlling growth and stress responses. *Biochem. J.* 474 2641–2661. 10.1042/bcj20160633 28751549 PMC6296487

[B102] Oh E. Zhu J. Y. Wang Z. Y. (2012). Interaction between BZR1 and PIF4 integrates brassinosteroid and environmental responses. *Nat. Cell Biol.* 14 802–809. 10.1038/ncb2545 22820378 PMC3703456

[B103] Oh K. Matsumoto T. Yamagami A. Hoshi T. Nakano T. Yoshizawa Y. (2015b). Fenarimol, a pyrimidine-type fungicide, inhibits brassinosteroid biosynthesis. *Int. J. Mol. Sci*. 16 17273–17288. 10.3390/ijms160817273 26230686 PMC4581192

[B104] Oh K. Matsumoto T. Yamagami A. Ogawa A. Yamada K. Suzuki R. (2015a). YCZ-18 is a new brassinosteroid biosynthesis inhibitor. *PLoS One*. 10:e0120812. 10.1371/journal.pone.0120812 25793645 PMC4368189

[B105] Oh M. H. Wang X. Kota U. Goshe M. B. Clouse S. D. Huber S. C. (2009). Tyrosine phosphorylation of the BRI1 receptor kinase emerges as a component of brassinosteroid signaling in *Arabidopsis*. *Proc. Natl. Acad. Sci.* 106 658–663. 10.1073/pnas.0810249106 19124768 PMC2613937

[B106] Oh M. H. Wang X. Wu X. Zhao Y. Clouse S. D. Huber S. C. (2010). Autophosphorylation of Tyr-610 in the receptor kinase BAK1 plays a role in brassinosteroid signaling and basal defense gene expression. *Proc. Natl. Acad. Sci. U. S. A.* 107 17827–17832. 10.1073/pnas.0915064107 20876109 PMC2955108

[B107] Ohnishi T. Godza B. Watanabe B. Fujioka S. Hategan L. Ide K. (2012). CYP90A1/CPD, a brassinosteroid biosynthetic cytochrome P450 of *Arabidopsis*, catalyzes C-3 oxidation. *J. Biol. Chem.* 287 31551–31560. 10.1074/jbc.m112.392720 22822057 PMC3438987

[B108] Ohri P. Bhardwaj R. Bali S. Kaur R. Jasrotia S. Khajuria A. (2015). The common molecular players in plant hormone crosstalk and signaling. *Curr. Prot. Pep. Sci.* 16 369–388. 10.2174/1389203716666150330141922 25824391

[B109] Ohri P. Bhardwaj R. Kaur R. Jasrotia S. Parihar R. D. Khajuria A. (2019). *Emerging Trends on Crosstalk of BRS with Other Phytohormones. In Brassinosteroids: Plant Growth and Development.* Singapore: Springer, 425–441.

[B110] Pál M. Csávás G. Szalai G. Oláh T. Khalil R. Yordanova R. (2017). Polyamines may influence phytochelatin synthesis during Cd stress in rice. *J. Hazardous Mater.* 340 272–280. 10.1016/j.jhazmat.2017.07.016 28715750

[B111] Paponov I. A. Teale W. D. Trebar M. Blilou I. Palme K. (2005). The PIN auxin efflux facilitators: evolutionary and functional perspectives. *Trends Plant Sci*. 10 170–177. 10.1016/j.tplants.2005.02.009 15817418

[B112] Planas-Riverola A. Gupta A. Betegón-Putze I. Bosch N. Ibañes M. Caño-Delgado A. I. (2019). Brassinosteroid signaling in plant development and adaptation to stress. *Development* 146:dev151894. 10.1242/dev.151894 30872266 PMC6432667

[B113] Peng Z. Han C. Yuan L. Zhang K. Huang H. Ren C. (2011). Brassinosteroid enhances jasmonate−induced anthocyanin accumulation in Arabidopsis seedlings. *J. Integr. Plant Bio.* 53 632–640. 10.1111/j.1744-7909.2011.01042.x 21545406

[B114] Peres A. L. G. Soares J. S. Tavares R. G. Righetto G. Zullo M. A. Mandava N. B. (2019). Brassinosteroids, the sixth class of phytohormones: a molecular view from the discovery to hormonal interactions in plant development and stress adaptation. *Int. J. Mol. Sci*. 20:331. 10.3390/ijms20020331 30650539 PMC6359644

[B115] Perilli S. Moubayidin L. Sabatini S. (2010). The molecular basis of cytokinin function. *Curr. Opin. Plant Bio*. 13 21–26. 10.1016/j.pbi.2009.09.018 19850510

[B116] Piotrowska-Niczyporuk A. Bajguz A. Zambrzycka E. Godlewska-Żyłkiewicz B. (2012). Phytohormones as regulators of heavy metal biosorption and toxicity in green alga Chlorella vulgaris (Chlorophyceae). *Plant Phys. Biochem*. 52 52–65. 10.1016/j.plaphy.2011.11.009 22305067

[B117] Polko J. K. Pierik R. van Zanten M. Tarkowská D. Strnad M. Voesenek L. A. (2013). Ethylene promotes hyponastic growth through interaction with ROTUNDIFOLIA3/CYP90C1 in Arabidopsis. *J. Exp. Bot.* 64 613–624. 10.1093/jxb/ers356 23264517 PMC3542051

[B118] Poppenberger B. Rozhon W. Khan M. Husar S. Adam G. Luschnig C. (2011). CESTA, a positive regulator of brassinosteroid biosynthesis. *EMBO J.* 30 1149–1161. 10.1038/emboj.2011.35 21336258 PMC3061039

[B119] Poonam R. K. Bali S. Singh R. Pati P. K. Bhardwaj R. (2014). Treatment of 24-EBL to *Brassica juncea* plants under Cu-metal stress lowers oxidative burst by activity antioxidative enzymes. *J. Stress Physiol. Biochem.* 10, 315–327.

[B120] Rattan A. Kapoor D. Kapoor N. Bhardwaj R. Sharma A. (2020). Brassinosteroids Regulate Functional Components of Antioxidative Defense System in Salt Stressed Maize Seedlings. *J. Plant Growth Reg.* 2020 1–11.

[B121] Rady M. M. (2011). Effect of 24-epibrassinolide on growth, yield, antioxidant system and cadmium content of bean (Phaseolus vulgaris L.) plants under salinity and cadmium stress. *Sci. Horticult.* 129 232–237. 10.1016/j.scienta.2011.03.035

[B122] Rady M. M. Osman A. S. (2012). Response of growth and antioxidant system of heavy metal-contaminated tomato plants to 24-epibrassinolide. *Afr. J. Agric. Res*. 7 3249–3254.

[B123] Rajewska I. Talarek M. Bajguz A. (2016). Brassinosteroids and response of plants to heavy metals action. *Front. Plant Sci.* 7:629. 10.3389/fpls.2016.00629 27242833 PMC4860867

[B124] Ramakrishna B. Rao S. S. R. (2015). Foliar application of brassinosteroids alleviates adverse effects of zinc toxicity in radish (*Raphanus sativus* L.) plants. *Protoplasma* 252 665–677. 10.1007/s00709-014-0714-0 25308099

[B125] Ross J. J. Quittenden L. J. (2016). Interactions between brassinosteroids and gibberellins: synthesis or signaling? *Plant Cell*. 28 829–832. 10.1105/tpc.15.00917 27006485 PMC4863384

[B126] Roychoudhury A. Ghosh S. Paul S. Mazumdar S. Das G. Das S. (2016). Pre-treatment of seeds with salicylic acid attenuates cadmium chloride-induced oxidative damages in the seedlings of mungbean (*Vigna radiata* L. Wilczek). *Acta Physiol. Planta*. 38:11.

[B127] Rozhon W. Mayerhofer J. Petutschnig E. Fujioka S. Jonak C. (2010). ASKtheta, a group-III Arabidopsis GSK3, functions in the brassinosteroid signalling pathway. *Plant J.* 62 215–223. 10.1111/j.1365-313x.2010.04145.x 20128883 PMC2881309

[B128] Rozhon W. Akter S. Fernandez A. Poppenberger B. (2019). Inhibitors of brassinosteroid biosynthesis and signal transduction. *Molecules* 24:4372. 10.3390/molecules24234372 31795392 PMC6930552

[B129] Saini S. Sharma I. Pati P. K. (2015). Versatile roles of brassinosteroid in plants in the context of its homoeostasis, signaling and crosstalks. *Front. Plant Sci.* 6:950. 10.3389/fpls.2015.00950 26583025 PMC4631823

[B130] Sharma I. Pati P. K. Bhardwaj R. (2011). Effect of 24-epibrassinolide on oxidative stress markers induced by nickel-ion in *Raphanus sativus L*. *Acta Physiol. Plant* 33, 1723–1735. 10.1007/s11738-010-0709-1

[B131] Sharma A. Thakur S. Kumar V. Kanwar M. K. Kesavan A. K. Thukral A. K. (2016). Pre-sowing seed treatment with 24-epibrassinolide ameliorates pesticide stress in *Brassica juncea* L. through the modulation of stress markers. *Front. Plant Sci.* 7:1569. 10.3389/fpls.2016.01569 PMC5089990 27853460

[B132] Sharma A. Thakur S. Kumar V. Kesavan A. K. Thukral A. K. Bhardwaj R. (2017). 24-epibrassinolide stimulates imidacloprid detoxification by modulating the gene expression of *Brassica juncea* L. *BMC Plant Bio.* 17:56. 10.1186/s12870-017-1003-9 PMC5477812 28245791

[B133] Shahzad B. Tanveer M. Che Z. Rehman A. Cheema S. A. Sharma A. (2018). Role of 24-epibrassinolide (EBL) in mediating heavy metal and pesticide induced oxidative stress in plants: A review. *Ecotoxico. Environ. Safety*. 147 935–944. 10.1016/j.ecoenv.2017.09.066 29029379

[B134] Sharma N. Hundal G. S. Sharma I. Bhardwaj R. (2014). 28-Homobrassinolide alters protein content and activities of glutathione-S-transferase and polyphenol oxidase in *Raphanus sativus* L. plants under heavy metal stress. *Toxico. Int*. 21:44. 10.4103/0971-6580.128792 PMC3989914 24748734

[B135] Sharma P. Kumar A. Bhardwaj R. (2016). Plant steroidal hormone epibrassinolide regulate–Heavy metal stress tolerance in *Oryza sativa* L. by modulating antioxidant defense expression. *Environ. Exp. Bot*. 122 1–9. 10.1016/j.envexpbot.2015.08.005

[B136] Singh S. Prasad S. M. (2017). Effects of 28-homobrassinoloid on key physiological attributes of *Solanum lycopersicum* seedlings under cadmium stress: photosynthesis and nitrogen metabolism. *Plant Growth Regul.* 82, 161–173. 10.1007/s10725-017-0248-5

[B137] Soares C. de Sousa A. Pinto A. Azenha M. Teixeira J. Azevedo R. A. (2016). Effect of 24-epibrassinolide on ROS content, antioxidant system, lipid peroxidation and Ni uptake in *Solanum nigrum* L. under Ni stress. *Environ. Exp. Bot*. 122 115–125. 10.1016/j.envexpbot.2015.09.010

[B138] Stewart Lilley J. L. Gan Y. Graham I. A. Nemhauser J. L. (2013). The effects of DELLA s on growth change with developmental stage and brassinosteroid levels. *Plant J.* 76 165–173. 23834248 10.1111/tpj.12280

[B139] Sun T. P. (2011). The molecular mechanism and evolution of the GA–GID1–DELLA signaling module in plants. *Curr. Bio*. 21 R338–R345. 21549956 10.1016/j.cub.2011.02.036

[B140] Sun Y. Fan X. Y. Cao D. M. Tang W. He K. Zhu J. Y. (2010). Integration of brassinosteroid signal transduction with the transcription network for plant growth regulation in *Arabidopsis*. *Dev. Cell.* 19 765–777. 10.1016/j.devcel.2010.10.010 21074725 PMC3018842

[B141] Symons G. M. Ross J. J. Jager C. E. Reid J. B. (2008). Brassinosteroid transport. *J. Exp. Bot.* 59 17–24. 10.1093/jxb/erm098 17709326

[B142] Szekeres M. Németh K. Koncz-Kálmán Z. Mathur J. Kauschmann A. Altmann T. (1996). Brassinosteroids rescue the deficiency of CYP90, a cytochrome P450, controlling cell elongation and de-etiolation in *Arabidopsis*. *Cell* 85 171–182. 10.1016/s0092-8674(00)81094-6 8612270

[B143] Tadayon M. S. Moafpourian G. (2019). Effects of Exogenous epi-brassinolid, zinc and boron foliar nutrition on fruit development and ripening of grape (*Vitis vinifera* L. clv.‘Khalili’). *Scient. Horticult.* 244 94–101. 10.1016/j.scienta.2018.09.036

[B144] Takahashi T. Kakehi J. I. (2010). Polyamines: ubiquitous polycations with unique roles in growth and stress responses. *Anna. Bot.* 105 1–6. 10.1093/aob/mcp259 19828463 PMC2794062

[B145] Tanaka K. Asami T. Yoshida S. Nakamura Y. Matsuo T. Okamoto S. (2005). Brassinosteroid homeostasis in Arabidopsis is ensured by feedback expressions of multiple genes involved in its metabolism. *Plant Physiol.* 138 1117–1125. 10.1104/pp.104.058040 15908602 PMC1150425

[B146] Tang W. Kim T. W. Oses-Prieto J. A. Sun Y. Deng Z. Zhu S. (2008). BSKs mediate signal transduction from the receptor kinase BRI1 in *Arabidopsis*. *Science* 321 557–560. 10.1126/science.1156973 18653891 PMC2730546

[B147] Tian H. Lv B. Ding T. Bai M. Ding Z. (2018). Auxin-BR interaction regulates plant growth and development. *Front. Plant Sci*. 8: 2256. 10.3389/fpls.2017.02256 PMC5778104 29403511

[B148] Tong H. Chu C. (2018). Functional specificities of brassinosteroid and potential utilization for crop improvement. *Trends Plant Sci*. 23 1016–1028. 10.1016/j.tplants.2018.08.007 30220494

[B149] Tong H. Xiao Y. Liu D. Gao S. Liu L. Yin Y. (2014). Brassinosteroid regulates cell elongation by modulating gibberellin metabolism in rice. *Plant Cell*. 26 4376–4393. 10.1105/tpc.114.132092 25371548 PMC4277228

[B150] Ulmasov T. Murfett J. Hagen G. Guilfoyle T. J. (1997). Aux/IAA proteins repress expression of reporter genes containing natural and highly active synthetic auxin response elements. *Plant Cell*. 9 1963–1971. 10.2307/3870557 9401121 PMC157050

[B151] Vert G. Walcher C. L. Chory J. Nemhauser J. L. (2008). Integration of auxin and brassinosteroid pathways by Auxin Response Factor 2. *Proc. Natl. Acad. Sci.* 105 9829–9834. 10.1073/pnas.0803996105 18599455 PMC2474533

[B152] Vriet C. Russinova E. Reuzeau C. (2012). Boosting crop yields with plant steroids. *Plant Cell*. 24 842–857. 10.1105/tpc.111.094912 22438020 PMC3336137

[B153] Vriet C. Russinova E. Reuzeau C. (2013). From squalene to brassinolide: the steroid metabolic and signaling pathways across the plant kingdom. *Mol. Plant*. 6 1738–1757. 10.1093/mp/sst096 23761349

[B154] Wang H. Tang J. Liu J. Hu J. Liu J. Chen Y. (2018). Abscisic acid signaling inhibits brassinosteroid signaling through dampening the dephosphorylation of BIN2 by ABI1 and ABI2. *Mol. Plant*. 11 315–325. 10.1016/j.molp.2017.12.013 29275167

[B155] Wang R. Wang J. Zhao L. Yang S. Song Y. (2015). Impact of heavy metal stresses on the growth and auxin homeostasis of *Arabidopsis* seedlings. *Biometals* 28 123–132. 10.1007/s10534-014-9808-6 25416404

[B156] Wang X. Chory J. (2006). Brassinosteroids regulate dissociation of BKI1, a negative regulator of BRI1 signaling, from the plasma membrane. *Science* 313 1118–1122. 10.1126/science.1127593 16857903

[B157] Wang X. Kota U. He K. Blackburn K. Li J. Goshe M. B. (2008). Sequential transphosphorylation of the BRI1/BAK1 receptor kinase complex impacts early events in brassinosteroid signaling. *Dev. Cell.* 15 220–235. 10.1016/j.devcel.2008.06.011 18694562

[B158] Wang Z. Y. Nakano T. Gendron J. He J. Chen M. Vafeados D. (2002). Nuclear-localized BZR1 mediates brassinosteroid-induced growth and feedback suppression of brassinosteroid biosynthesis. *Dev. Cell*. 2 505–513. 10.1016/s1534-5807(02)00153-3 11970900

[B159] Watanabe T. Noguchi T. Yokota T. Shibata K. Koshino H. Seto H. (2001). Synthesis and biological activity of 26-norbrassinolide, 26-norcastasterone and 26-nor-6-deoxocastasterone. *Phytochem* 58 343–349. 10.1016/s0031-9422(01)00213-8 11551562

[B160] Werner T. Schmülling T. (2009). Cytokinin action in plant development. *Curr. Opin. Plant Bio*. 12 527–538. 19740698 10.1016/j.pbi.2009.07.002

[B161] Wu C. Li F. Xu H. Zeng W. Yu R. Wu X. (2019). The potential role of brassinosteroids (BRs) in alleviating antimony (Sb) stress in *Arabidopsis thaliana*. *Plant Physio Biochem*. 141 51–59. 10.1016/j.plaphy.2019.05.011 31128563

[B162] Xia X. J. Fang P. P. Guo X. Qian X. J. Zhou J. Shi K. (2018). Brassinosteroid−mediated apoplastic H2O2−glutaredoxin 12/14 cascade regulates antioxidant capacity in response to chilling in tomato. *Plant. Cell ENV*. 41 1052–1064. 28776692 10.1111/pce.13052

[B163] Xia X. J. Gao C. J. Song L. X. Zhou Y. H. Shi K. A. I. Yu J. Q. (2014). Role of H2O2 dynamics in brassinosteroid−induced stomatal closure and opening in *Solanum lycopersicum*. *Plant. Cell ENV*. 37 2036–2050. 24428600 10.1111/pce.12275

[B164] Xia X. J. Zhou Y. H. Shi K. Zhou J. Foyer C. H. Yu J. Q. (2015). Interplay between reactive oxygen species and hormones in the control of plant development and stress tolerance. *J. Exp. Bot.* 66 2839–2856. 25788732 10.1093/jxb/erv089

[B165] Yamamuro C. Ihara Y. Wu X. Noguchi T. Fujioka S. Takatsuto S. (2000). Loss of function of a rice brassinosteroid insensitive1 homolog prevents internode elongation and bending of the lamina joint. *Plant Cell.* 12 1591–1605. 11006334 10.1105/tpc.12.9.1591 PMC149072

[B166] Yan J. Zhang C. Gu M. Bai Z. Zhang W. Qi T. (2009). The Arabidopsis CORONATINE INSENSITIVE1 protein is a jasmonate receptor. *Plant Cell* 21, 2220–2236. 10.1105/tpc.109.065730 19717617 PMC2751961

[B167] Ye H. Li L. Guo H. Yin Y. (2012). MYBL2 is a substrate of GSK3-like kinase BIN2 and acts as a corepressor of BES1 in brassinosteroid signaling pathway in *Arabidopsis*. *Proc. Natl. Acad. Sci*. 109 20142–20147. 23169658 10.1073/pnas.1205232109 PMC3523856

[B168] Yin Y. L. Zhou Y. Zhou Y. H. Shi K. Zhou J. Yu Y. (2016). Interplay between mitogen-activated protein kinase and nitric oxide in brassinosteroid-induced pesticide metabolism in *Solanum lycopersicum*. *J. Hazard. Mater.* 316 221–231. 27236431 10.1016/j.jhazmat.2016.04.070

[B169] Yin Y. Qin K. Song X. Zhang Q. Zhou Y. Xia X. (2018). BZR1 transcription factor regulates heat stress tolerance through FERONIA receptor-like kinase-mediated reactive oxygen species signaling in tomato. *Plant Cell Physio*. 59 2239–2254. 10.1093/pcp/pcy146 30107607

[B170] Yin Y. Vafeados D. Tao Y. Yoshida S. Asami T. Chory J. (2005). A new class of transcription factors mediates brassinosteroid regulated gene expression in *Arabidopsis*. *Cell* 120 249–259. 15680330 10.1016/j.cell.2004.11.044

[B171] Yokota T. (1999). The history of brassinosteroids: discovery to isolation of biosynthesis and signal transduction mutants. *Brassinosteroids* 1999 1–20.

[B172] Yokota T. Ogino Y. Suzuki H. Takahashi N. Saimoto H. Fujioka S. (1991). Metabolism and biosynthesis of brassinosteroids. In: Brassinosteroids: Chemistry. *Bioact. Appl.* 1991 86–96.

[B173] Yoshimitsu Y. Tanaka K. Fukuda W. Asami T. Yoshida S. Hayashi K. I. (2011). Transcription of DWARF4 plays a crucial role in auxin-regulated root elongation in addition to brassinosteroid homeostasis in *Arabidopsis thaliana*. *PLoS One*. 6:e23851. 10.1371/journal.pone.0023851 PMC3166115 21909364

[B174] Yu X. Li L. Zola J. Aluru M. Ye H. Foudree A. (2011). A brassinosteroid transcriptional network revealed by genome−wide identification of BESI target genes in *Arabidopsis thaliana*. *Plant J.* 65 634–646. 21214652 10.1111/j.1365-313X.2010.04449.x

[B175] Yuan L. B. Peng Z. H. Zhi T. T. Zho Z. Liu Y. Zhu Q. (2014). Brassinosteroid enhances cytokinin-induced anthocyanin biosynthesis in *Arabidopsis* seedlings. *Biol. Plant.* 59 99–105.

[B176] Yuldashev R. Avalbaev A. Bezrukova M. Vysotskaya L. Khripach V. Shakirova F. (2012). Cytokinin oxidase is involved in the regulation of cytokinin content by 24-epibrassinolide in wheat seedlings. *Plant Physio Biochem*. 55 1–6. 10.1016/j.plaphy.2012.03.004 22480990

[B177] Yunta C. Martínez-Ripoll M. Zhu J. K. Albert A. (2011). The structure of Arabidopsis thaliana OST1 provides insights into the kinase regulation mechanism in response to osmotic stress. *J. Mol. Bio*. 414 135–144. 21983340 10.1016/j.jmb.2011.09.041 PMC3593245

[B178] Yusuf M. Fariduddin Q. Ahmad A. (2012). 24-Epibrassinolide modulates growth, nodulation, antioxidant system, and osmolyte in tolerant and sensitive varieties of *Vigna radiata* under different levels of nickel: a shotgun approach. *Plant Physio Biochem*. 57 143–153. 10.1016/j.plaphy.2012.05.004 22705589

[B179] Yusuf M. Fariduddin Q. Hayat S. Hasan S. A. Ahmad A. (2011). Protective response of 28-homobrassinolide in cultivars of *Triticum aestivum* with different levels of nickel. *Arch. Environ. Cont. Toxico*. 60 68–76. 10.1007/s00244-010-9535-0 20464550

[B180] Zhang A. Zhang J. Zhang J. Ye N. Zhang H. Tan M. (2011). Nitric oxide mediates brassinosteroid-induced ABA biosynthesis involved in oxidative stress tolerance in maize leaves. *Plant Cell Physio*. 52 181–192. 10.1093/pcp/pcq187 21134899

[B181] Zhang S. Cai Z. Wang X. (2009). The primary signaling outputs of brassinosteroids are regulated by abscisic acid signaling. *Proc. Natl. Acad. Sci.* 106 4543–4548. 19240210 10.1073/pnas.0900349106 PMC2657416

[B182] Zhang X. Guo W. Du D. Pu L. Zhang C. (2020). Overexpression of a maize BR transcription factor ZmBZR1 in *Arabidopsis* enlarges organ and seed size of the transgenic plants. *Plant Sci*. 292: 110378. 10.1016/j.plantsci.2019.110378 32005383

[B183] Zhao B. Li J. (2012). Regulation of brassinosteroid biosynthesis and inactivation F. *J. Integr. Plant Biol.* 54 746–759. 22963251 10.1111/j.1744-7909.2012.01168.x

[B184] Zhou Y. L. Huo S. F. Wang L. T. Meng J. F. Zhang Z. W. Xi Z. M. (2018). Exogenous 24-Epibrassinolide alleviates oxidative damage from copper stress in grape (Vitis vinifera L.) cuttings. *Plant Physio. Biochem.* 130 555–565. 10.1016/j.plaphy.2018.07.029 30099273

[B185] Zhu T. Tan W. R. Deng X. G. Zheng T. Zhang D. W. Lin H. H. (2015). Effects of brassinosteroids on quality attributes and ethylene synthesis in postharvest tomato fruit. *Postharvest Bio Techno*. 100 196–204.

[B186] Zou L. Qu M. Zeng L. Xiong G. (2020). The molecular basis of the interaction between Brassinosteroid induced and phosphorous deficiency induced leaf inclination in rice. *Plant Growth Regul*. 2020 1–14.

[B187] Zullo M. A. T. (2018). *Brassinosteroids and related compounds.* New York: LAP LAMBERT Academic Publishing.

[B188] Zullo M. A. T. Adam G. (2002). Brassinosteroid phytohormones: structure, bioactivity and applications. *Braz. J. Plant Physiol.* 14 143–181.

[B189] Zullo M. A. T. Bajguz A. (2019). *The brassinosteroids family–structural diversity of natural compounds and their precursors. In Brassinosteroids: Plant Growth and Dev.* Singapore: Springer, 1–44.

[B190] Zullo M. A. T. Kohout L. (2004). Semisystematic nomenclature of brassinosteroids. *Plant Growth Regul.* 42 15–28.

